# Formate overflow drives toxic folate trapping in MTHFD1 inhibited cancer cells

**DOI:** 10.1038/s42255-023-00771-5

**Published:** 2023-04-03

**Authors:** Alanna C. Green, Petra Marttila, Nicole Kiweler, Christina Chalkiadaki, Elisée Wiita, Victoria Cookson, Antoine Lesur, Kim Eiden, François Bernardin, Karl S. A. Vallin, Sanjay Borhade, Maeve Long, Elahe Kamali Ghahe, Julio J. Jiménez-Alonso, Ann-Sofie Jemth, Olga Loseva, Oliver Mortusewicz, Marianne Meyers, Elodie Viry, Annika I. Johansson, Ondřej Hodek, Evert Homan, Nadilly Bonagas, Louise Ramos, Lars Sandberg, Morten Frödin, Etienne Moussay, Ana Slipicevic, Elisabeth Letellier, Jérôme Paggetti, Claus Storgaard Sørensen, Thomas Helleday, Martin Henriksson, Johannes Meiser

**Affiliations:** 1 Weston Park Cancer Centre and Mellanby Centre for Musculoskeletal Research, Department of Oncology and Metabolism, The Medical School, University of Sheffield, Sheffield, UK; 2 Science for Life Laboratory, Department of Oncology-Pathology, Karolinska Institutet, Solna, Sweden; 3 Cancer Metabolism Group, Department of Cancer Research, Luxembourg Institute of Health, Luxembourg, Luxembourg; 4 RISE Research Institutes of Sweden, Södertälje, Sweden; 5 RedGlead Discover, Lund, Sweden; 6 Biotech Research and Innovation Centre, University of Copenhagen, Copenhagen, Denmark; 7 Department of Pharmacology, Faculty of Pharmacy, University of Seville, Seville, Spain; 8 Faculty of Science, Technology and Medicine, Department of Life Sciences and Medicine, Molecular Disease Mechanisms Group, University of Luxembourg, Esch-sur-Alzette, Luxembourg; 9 Tumor Stroma Interactions, Department of Cancer Research, Luxembourg Institute of Health, Luxembourg, Luxembourg; 10 Swedish Metabolomics Centre, Department of Plant Physiology, Umeå University, Umeå, Sweden; 11 Department of Forest Genetics and Plant Physiology, Swedish University of Agricultural Sciences, Umeå, Sweden; 12 Drug Discovery and Development Platform, Science for Life Laboratory, Department of Organic Chemistry, Stockholm University, Solna, Sweden; 13 One-carbon Therapeutics AB, Stockholm, Sweden

**Keywords:** Cancer metabolism, Cell death, Metabolism

## Abstract

Cancer cells fuel their increased need for nucleotide supply by upregulating one-carbon (1C) metabolism, including the enzymes methylenetetrahydrofolate dehydrogenase–cyclohydrolase 1 and 2 (MTHFD1 and MTHFD2). TH9619 is a potent inhibitor of dehydrogenase and cyclohydrolase activities in both MTHFD1 and MTHFD2, and selectively kills cancer cells. Here, we reveal that, in cells, TH9619 targets nuclear MTHFD2 but does not inhibit mitochondrial MTHFD2. Hence, overflow of formate from mitochondria continues in the presence of TH9619. TH9619 inhibits the activity of MTHFD1 occurring downstream of mitochondrial formate release, leading to the accumulation of 10-formyl-tetrahydrofolate, which we term a ‘folate trap’. This results in thymidylate depletion and death of MTHFD2-expressing cancer cells. This previously uncharacterized folate trapping mechanism is exacerbated by physiological hypoxanthine levels that block the de novo purine synthesis pathway, and additionally prevent 10-formyl-tetrahydrofolate consumption for purine synthesis. The folate trapping mechanism described here for TH9619 differs from other MTHFD1/2 inhibitors and antifolates. Thus, our findings uncover an approach to attack cancer and reveal a regulatory mechanism in 1C metabolism.

## Abstract (web summary)

In this study, Green, Marttila, Kiweler et al. characterize one-carbon metabolism rewiring in response to a dual MTHFD1 and MTHFD2 inhibitor. This work provides insight into one-carbon fluxes, and reveals a previously uncharacterized vulnerability in cancer cells created by folate trapping.

## Main

One-carbon (1C) metabolism is central to the supply of nucleotides for DNA synthesis and repair. Without sufficient nucleotide supply to meet the proliferative demands, cells undergo cell cycle arrest or cell death due to replication stress and genomic instability^1–3^. Enzymes involved in the 1C metabolism pathway are commonly upregulated in cancer to produce nucleotides and amino acids required for rapid proliferation^4–6^.

**Fig. 1:**
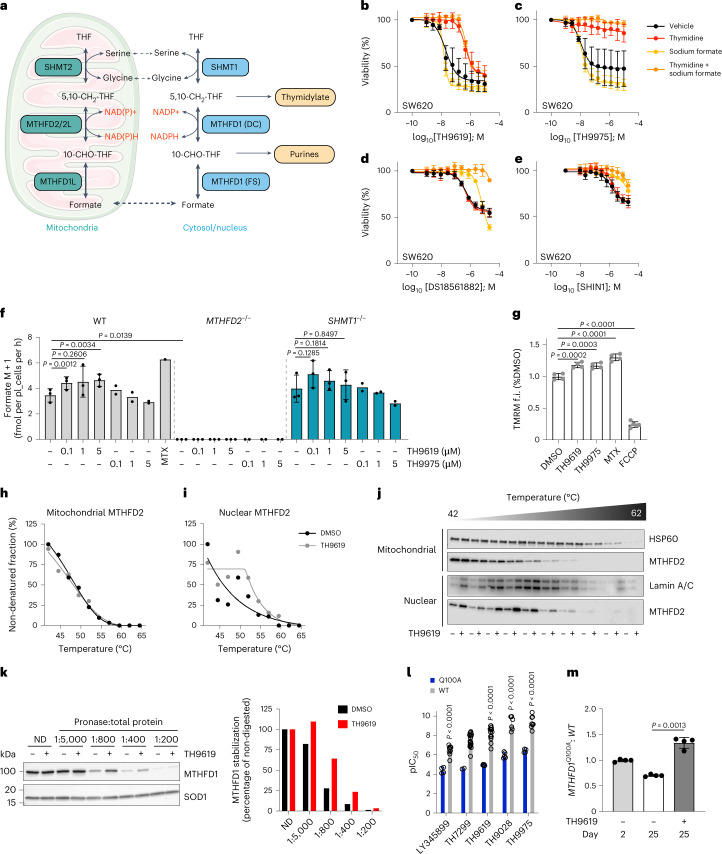
TH9619 and TH9975 target MTHFD1(DC) but not mitochondrial MTHFD2. **a**, 1C metabolism flux between mitochondria and cytosol/nucleus. **b**–**e**, Dose–response curves of SW620 cells treated for 96 h with TH9619 (**b**), TH9975 (**c**), DS18561882 (**d**) or SHIN1 (**e**) in the presence of 50 μM thymidine, 1 mM sodium formate or vehicle (cultured in RPMI-FBS), means ± s.d. (*n* = 3). **f**, [U-^13^C]serine-derived formate release rate of SW620 WT, *MTHFD2*^*−/−*^ and *SHMT1*^*−/−*^ cells treated for 24 h with the indicated concentrations of TH9619 and TH9975 or 50 nM MTX (cultured in RPMI-FBS), means ± s.d. (*n* = 3 for control and TH9619, *n* = 2 for TH9975, *n* = 1 for MTX); one-way ANOVA (analysis of variance) with Tukey’s multiple comparisons test. **g**, Quantification of mitochondrial membrane potential in SW620 WT cells treated with 1 µM of TH9619 or TH9975 or 0.5 µM MTX for 48 h. FCCP was used as positive control. Data are displayed as means ± s.d. (*n* = 4, *n* = 5 for FCCP); one-way ANOVA with Dunnett’s test for multiple comparisons. **h**,**i**, CETSA analysis of MTHFD2 stabilization in SW620 WT cells treated for 3 h with 10 µM TH9619 or vehicle. MTHFD2 stabilization was assessed by western blot by normalizing remaining MTHFD2 signal to HSP60 in the mitochondrial fractions (**h**) or lamin A/C in the nuclear fractions (**i**). **j**, Representative immunoblots of one out of two independent experiments. **k**, DARTS analysis of MTHFD1 stabilization in SW620 lysates that were incubated with 10 µM TH9619 or vehicle for 30 min, followed by protein digestion at increasing pronase concentration and assessment of MTHFD1 degradation by western blot. MTHFD1 signal was normalized to SOD1. Shown is a representative out of three independent experiments. **l**, Mean pIC_50_ (−log of half-maximum inhibitory concentration) values for indicated MTHFD1/2 inhibitors assessed for their inhibitory activity against MTHFD1(DC) WT or MTHFD1(DC) mutant (Q100A), mean (from left to right *n* = 4, 9, 2, 19, 5, 15, 5, 6, 4, 8); unpaired, two-tailed *t*-test. **m**, CRISPR-Select cassette for MTHFD1-Q100A was delivered to SW620 cells. The graph shows the ratio of MTHFD1-Q100A versus WT on day 2 and day 25 of 10 μM TH9619 treatment normalized to day 2 value, means ± s.d. (*n* = 4); paired, two-tailed *t*-test. Source data

The 1C metabolism is predominantly fuelled by the non-essential amino acid serine, which can be generated from the glycolytic intermediate 3-phosphoglycerate or supplied from the extracellular space^7^. Catabolism of serine via folate-dependent 1C metabolism is required for nucleotide synthesis. It is mediated by four reversible enzymatic reactions that occur in the mitochondria and cytosol (Fig. 1a). Serine hydroxymethyl transferase (SHMT) transfers a hydroxymethyl group from serine to tetrahydrofolate (THF) to generate 5,10-methylenetetrahydrofolate (CH_2_-THF) and glycine. Methylenetetrahydrofolate dehydrogenase–cyclohydrolase (MTHFD) oxidizes and hydrolyses CH_2_-THF to 10-formyl-tetrahydrofolate (10-CHO-THF), which is then hydrolysed to THF and formate by formyl THF synthetase. In the cytosol, these reactions are catalysed by SHMT1 and the tri-functional MTHFD1, which comprises two distinct domains, the dehydrogenase–cyclohydrolase (DC) domain and the formyl THF synthetase (FS) domain. In the mitochondrion, the same reactions are catalysed by SHMT2, bifunctional MTHFD2 (DC activity) and MTHFD1L (FS activity) (Fig. 1a)^8^. Although variations exist^9^, the canonical directionality of the pathway follows serine catabolism and 1C unit oxidation via the mitochondrion, while cytosolic 1C units follow a reductive (reverse) route that is driven by a high cytosolic NADPH:NADP^+^ ratio^8,10–12^. This way, the 1C metabolism forms a cycle that is spread between mitochondrion and cytoplasm. Due to the reversibility of the SHMT1 and MTHFD1 catalysed reactions, loss of mitochondrial 1C metabolism can be compensated via the cytosolic route^13^.

Recently, the mitochondrial enzyme MTHFD2 has gained increasing attention, as it has been identified as one of the most highly upregulated genes in cancer^6,14,15^. A large body of validation work of MTHFD2 as an anticancer target has been ongoing in recent years, demonstrating that MTHFD2 RNA interference depletion or inhibition can kill cancer cells of diverse origin such as acute myeloid leukaemia (AML)^16,17^, breast cancer^18^ and colorectal cancer^19^. This work contrasts CRISPR–Cas9 knockout results demonstrating *MTHFD2*^−/−^ cancer cell lines are mostly alive^20^, and further research is required to understand these discrepancies.

We recently developed nanomolar, small-molecule MTHFD2 inhibitors (MTHFD2i). Owing to similar active sites in both enzymes, the inhibitors also target the DC domain of MTHFD1 (ref. ^16^). Here, we expand on our previous findings and revisit the mechanism of action of TH9619, and find that the compound does not enter mitochondria to target mitochondrial MTHFD2. Instead, we show that TH9619 targets MTHFD2-expressing cancer cells by inhibiting MTHFD1(DC). This phenomenon occurs because TH9619 requires MTHFD2 activity to cause thymidylate deficiency and cell toxicity. TH9619 inhibition of MTHFD1(DC) causes accumulation of the 1C intermediate 10-CHO-THF in MTHFD2-expressing cancer cells (a mechanism we term ‘folate trapping’). The subsequent depletion of free cytosolic THF starves SHMT1 of its substrate and prevents downstream thymidylate synthesis. Moreover, we show that the purine-derivative hypoxanthine is a strong potentiator of this mechanism. Hypoxanthine contributes to folate trapping through exogenous supply of purines, providing feedback inhibition on de novo purine synthesis. Without consumption of 10-CHO-THF for de novo purine synthesis, folate trapping is potentiated and thymidylate synthesis is blocked. In addition to its toxicity in MTHFD2-expressing cancers, we also discover that TH9619 has a potent antitumorigenic effect on cancers deficient in mitochondrial 1C enzymes (MTHFD2 and MTHFD1L) by blocking purine synthesis. In summary, we detail the complex metabolic pathway that contributes to efficient cancer-specific killing with TH9619, a prerequisite to translate TH9619 into the clinic. In wider terms, the obtained knowledge vastly improves our understanding of how to effectively target 1C metabolism to induce cytotoxicity in cancer cells.

## Results

### TH9619/TH9975 target MTHFD1(DC) but not mitochondrial MTHFD2

In our recent study, we demonstrated that loss of MTHFD2 caused resistance to TH9619, validating MTHFD2 as a target of TH9619 (ref. ^16^). Yet, we showed that the effect of TH9619 is metabolically rescued by addition of thymidine, but not with hypoxanthine. This was surprising given that SHMT1 can support thymidylate synthesis independently of the MTHFD enzymes^13^. Thus, we wanted to further investigate whether TH9619 directly targets MTHFD2 and/or MTHFD1 with the aim to better understand the mode of action of MTHFD1/2i.

**Extended Data Fig. 1:**
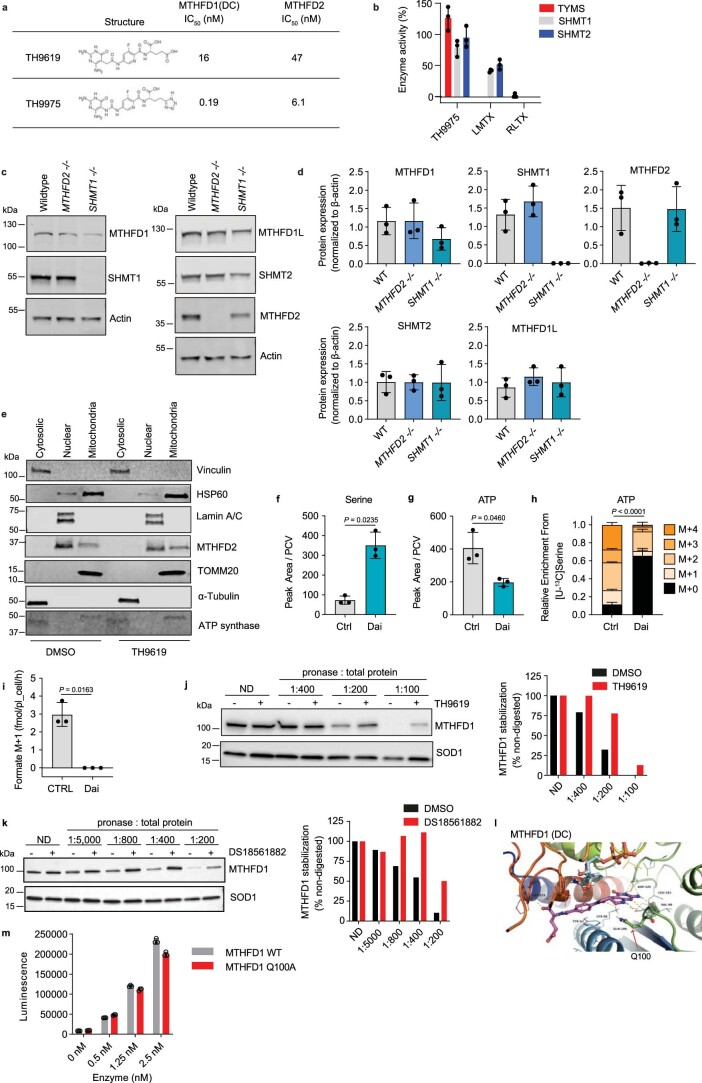
TH9619 and TH9975 are inhibitors of MTHFD1(DC) *in vitro*. (**a**) Chemical structures of MTHFD1/2 inhibitors TH9619 and TH9975 and IC_50_ values for inhibition of MTHFD1(DC) and MTHFD2 enzymatic activities for each inhibitor. (**b**) Enzymatic activity of TYMS, SHMT1 and SHMT2 upon incubation with 100 µM TH9975 compared to their known inhibitors lometrexol (LMTX) and raltitrexed (RLTX), means ± SD (n = 3). (**c**, **d**) Expression of the indicated proteins was analyzed by Western Blot in SW620 WT, *MTHFD2*^*-/-*^ and *SHMT1*^*-/-*^ cells. β-actin served as loading control. Immunoblots are representative of 3 independent repeats. Signal intensity was quantified and normalized to loading control and global mean, means ± SD (n = 3). (**e**) Verification of the purity of mitochondrial and nuclear fractions for CETSA analysis. Shown are representative immunoblots of one out of two independent experiments. (**f**, **g**) Serine and ATP levels normalized to packed cell volume (PCV) in SW620 WT cells treated for 24 h with 10 µM DS18561882, mean ± SD (n = 3); paired, two-tailed t-test. (**h**) [U-^13^C]serine-derived MID of ATP in SW620 WT cells treated for 24 h with 10 µM DS18561882, mean ± SD (n = 3); one-way ANOVA with Šídák’s multiple comparison test for M + 0. (**i**) [U-^13^C]serine-derived release rate of formate M + 1 isotopologue from SW620 WT cells treated for 24 h with 10 µM DS18561882, mean ± SD (n = 3); paired, two-tailed t-test. (**j**, **k**) DARTS analysis of MTHFD1 stabilization in SW620 cells upon TH9619 (**j**) or DS18561882 (**k**) treatment. Intact cells (**j**) or cell lysates (**k**) were incubated with 10 µM TH9619 or vehicle for 3 h (**j**) or 30 min (**k**), followed by protein digestion at increasing pronase concentration and assessment of MTHFD1 degradation by Western blot. MTHFD1 intensities were normalized to SOD1; one representative immunoblot of two independent experiments. (**l**) Design of MTHFD1/2 inhibitor resistant MTHFD1 protein based on the crystal structure of structurally similar MTHFD2 bound to LY345899 (PDB ID: 5TC4). Highlighted is the residue Q100 that was mutated to alanine (Q100A) and tested in downstream biochemical assays. (**m**) Enzymatic activity of purified MTHFD1(DC) WT and MTHFD1(DC) mutant (Q100A), means ± SD (n = 3). Source data

MTHFD2 and MTHFD1L normally produce formate in the mitochondria, which is then consumed by cytosolic 1C metabolism to produce thymidylate and purines (Fig. 1a)^13^. If TH9619 inhibits mitochondrial MTHFD2, this would block mitochondrial production of formate, and presumably exogenous addition of formate should overcome inhibitor activity. However, addition of 1 mM sodium formate did not rescue viability on TH9619 treatment, and had no additional effect when combined with thymidine, indicating formate depletion is not responsible for TH9619 toxicity (Fig. 1b). Similarly, formate did not rescue the effect of TH9975 (Fig. 1c), another MTHFD1/2i from the same series (Extended Data Fig. 1a,b). This contrasts with the previously described 1C metabolism inhibitors DS18561882 (ref. ^18^) (MTHFD1/2i, ref. ^16^) and SHIN1 (ref. ^21^) (SHMT1/2i), which were both partially rescued by addition of sodium formate (Fig. 1d,e).

We previously demonstrated that cancer cells generate formate in excess of the anabolic demand resulting in formate excretion (formate overflow)^22,23^. This formate overflow specifically depends on active mitochondrial 1C metabolism^24^. Thus, formate overflow can be used as a readout to assess the activity of mitochondrial 1C metabolism. To investigate this further, formate release rates from cells were measured by gas chromatography–MS. As expected, *MTHFD2*^*−/−*^ cells had no formate release (Fig. 1f). In contrast, TH9619 and TH9975 did not impair formate release in wild-type (WT) or CRISPR–Cas9 generated *SHMT1*^*−/−*^ SW620 cells (Fig. 1f and Extended Data Fig. 1c,d). In fact, TH9619 increased formate release rates of SW620 WT, most probably as a result of MTHFD1(DC) inhibition that prevents thymidylate synthesis and recycling of mitochondrial-derived formate. A similar phenomenon has previously been reported for methotrexate (MTX), which inhibits dihydrofolate reductase but does not impair mitochondrial 1C metabolism^24^, and served as a positive control in our experimental setup (Fig. 1f). Furthermore, mitochondrial membrane potential was not impaired, but TH9619 and TH9975 caused a slight hyperpolarization after 48 h (Fig. 1g). This hyperpolarization may be a secondary effect, caused by small changes in nucleotide balances, rather than direct effects of TH9619 and TH9975 on mitochondrial function. Together, this indicates that TH9619 and TH9975 are not inhibiting mitochondrial MTHFD2.

The metabolic data and the functional assay indicating that TH9619 does not directly inhibit MTHFD2 (Fig. 1b–g) contrast with our previous study showing that TH9619 binds and stabilizes MTHFD2 in whole cells and cell lysates from leukaemic HL-60 cells using a cellular thermal shift assay (CETSA)^16^. MTHFD2 is located both in the mitochondria and in the nucleus^25^, meaning that the stabilization we observed could be due to MTHFD2 stabilization in either of these organelles. Therefore, we performed CETSA on purified subcellular fractions of mitochondria and nuclei (Extended Data Fig. 1e). Indeed, while TH9619 stabilized nuclear MTHFD2, there was no stabilization of mitochondrial MTHFD2 (Fig. 1h–j), supporting the notion that TH9619 does not inhibit mitochondrial MTHFD2. Since its physicochemical properties prohibit passive membrane permeation, we propose that TH9619 is not a substrate for the mitochondrial folate transporter (SLC25A32) and hence cannot enter the mitochondria to engage with MTHFD2. In contrast, DS18561882 inhibits formate release and ATP biosynthesis, while increasing intracellular serine levels, indicating mitochondrial MTHFD2 inhibition (Extended Data Fig. 1f–i). DS18561882 is much more lipophilic than TH9619 and likely to passively diffuse across membranes, thus not requiring SLC25A32 to reach mitochondrial MTHFD2.

Owing to technical limitations, we had not previously been able to determine whether TH9619 binds to MTHFD1 in cells by CETSA. Here, we used a drug affinity responsive target stability (DARTS)^26^ assay and found that TH9619 was able to stabilize MTHFD1 in lysates and whole cells (Fig. 1k and Extended Data Fig. 1j), suggesting MTHFD1 may play a role in TH9619 toxicity despite of a lack of resistance in *MTHFD1*^*KD*^ cells. We also found that DS18561882 (MTHFD1/2i) engaged MTHFD1 in lysates (Extended Data Fig. 1k). To explore whether targeting MTHFD1 is important for toxicity, we generated a MTHFD1(DC) mutant that is resistant to TH9619 and other compounds in this series (TH7299, ref. ^16^; TH9028, ref. ^16^ and TH9975) but is still enzymatically active. Using the crystal structure of MTHFD2 bound to an inhibitor (TH7299)^16^, which resembles MTHFD1(DC), we predicted that exchanging glutamine for an alanine at position 100 (Q100A) would drastically reduce the affinity for MTHFD1/2i, without majorly affecting the catalytic activity of the enzyme (Extended Data Fig. 1l). Hence, this could prompt a resistance mutation to the drugs. To test this, we purified *MTHFD1*^*Q100A*^ and determined its biochemical activity. While the mutant showed comparable enzymatic activity to the WT enzyme (Extended Data Fig. 1m), we found, as expected, that the Q100A mutation led to an increased resistance to all the inhibitors in the series (Fig. 1l). To confirm involvement of MTHFD1, we used the recently developed technique CRISPR-Select^27^, to introduce the *MTHFD1*^*Q100A*^ mutation into cells. *MTHFD1*^*Q100A*^ cells accumulated roughly 1.9-fold compared to WT cells in the cell population over the 25-day experiment (Fig. 1m), demonstrating that the Q100A mutation increases cell proliferation and confers partial resistance to TH9619. This indicates that toxicity to TH9619 is at least partially due to targeting of MTHFD1(DC).

Together, these data along with our previous findings^16^, reveal that both MTHFD1(DC) and MTHFD2 are required for TH9619 mechanism of action, but that the drug toxicity is not due to TH9619 directly targeting mitochondrial MTHFD2. Given the surmounting evidence for a potentially non-catalytic role of MTHFD2 in the nucleus^25,28^, and for a stabilization of nuclear MTHFD2 by TH9619, TH9619 may also play a role in regulating nuclear MTHFD2 activity at replication forks. Whereas this role merits further investigation in future studies, here we focus on the metabolic effects of 1C metabolism to explain the potent, cancer-specific toxicity of TH9619.

### TH9619 primarily inhibits thymidylate synthesis in WT cells

The 1C units for purine and thymidylate synthesis can be generated from serine by cytosolic or mitochondrial 1C metabolism (Fig. 1a)^13^. Given that MTHFD2 expression sensitizes to TH9619, but that mitochondrial MTHFD2 is not directly inhibited and that TH9619 also targets MTHFD1(DC), we further investigated the impact of TH9619 on thymidylate and purine synthesis. We performed metabolic rescue experiments with thymidine, hypoxanthine, folinic acid and folic acid, and compared TH9619 to TH9975 (MTHFD1/2i), DS18561882 (ref. ^18^) (MTHFD1/2i, ref. ^16^), SHIN1^21^ (SHMT1/2i), MTX and 5-fluorouracil (5-FU).

**Fig. 2:**
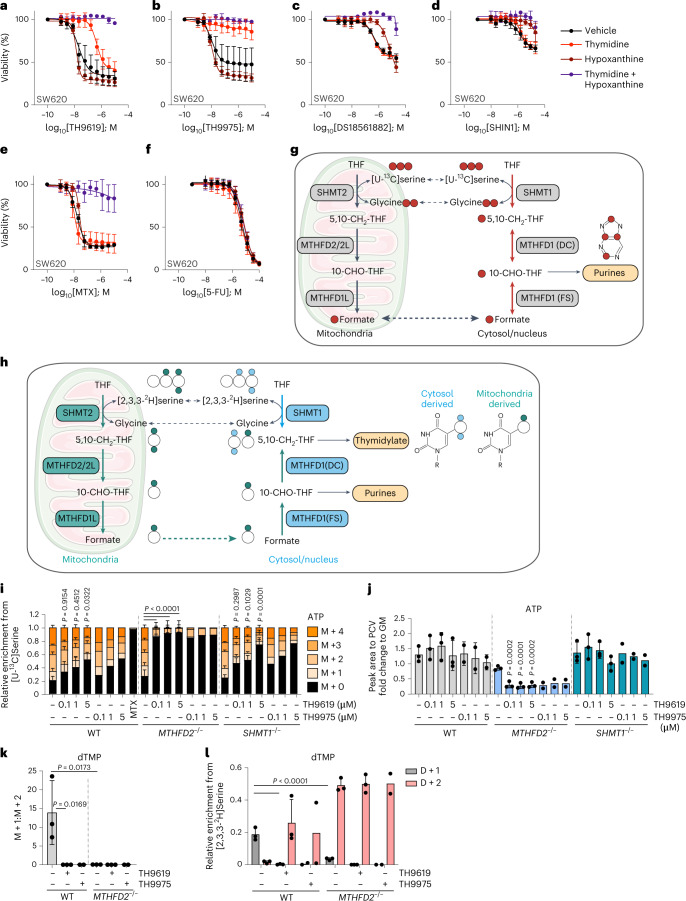
TH9619 primarily inhibits thymidylate biosynthesis in SW620 WT cells and purine biosynthesis in *MTHFD2*^*−/−*^ cells. **a**–**f**, Dose–response curves of SW620 WT cells treated for 96 h with TH9619 (**a**), TH9975 (**b**), DS18561882 (**c**), SHIN1 (**d**), MTX (**e**) or 5-FU (**f**) in the presence of 50 μM thymidine, 250 μM hypoxanthine or vehicle (cultured in RPMI-FBS), means ± s.d. (*n* = 3). **g**, 1C metabolism labelling derived from [U-^13^C] serine tracer with red dots indicating ^13^C atoms in individual metabolites. 5,10-CH_2_-THF, 5,10-methylenetetrahydrofolate; 10-CHO-THF, 10-formyl tetrahydrofolate; MTHFD1/1L/2/2L, methylenetetrahydrofolate dehydrogenase 1/1L/2/2L. **h**, 1C metabolism labelling derived from [2,3,3-^2^H]serine tracer with coloured dots indicating cytosol and mitochondrial-derived ^2^H atoms in individual metabolites. **i**, [U-^13^C]serine-derived MID of ATP in SW620 WT, *MTHFD2*^*−/−*^ and *SHMT1*^*−/−*^ cells treated for 24 h with the indicated concentrations of TH9619 and TH9975 in μM or 50 nM MTX (cultured in RPMI-FBS), mean ± s.d. (*n* = 3 for untreated and TH9619, *n* = 2 for TH9975, *n* = 1 for MTX); one-way ANOVA with Tukey’s multiple comparisons test for M + 0. *P* values indicate comparisons to untreated control of each cell line. **j**, ATP level normalized to PCV in SW620 WT, *MTHFD2*^*−/−*^ and *SHMT1*^*−/−*^ cells treated for 24 h with the indicated concentrations of TH9619 and TH9975 in μM (cultured in RPMI-FBS), means ± s.d. (*n* = 3 for untreated and TH9619, *n* = 2 for TH9975); one-way ANOVA with Dunnett’s test for multiple comparisons. *P* values indicate comparisons to the untreated control. **k**, Ratio of the [2,3,3-^2^H]serine-derived M + 1 to M + 2 isotopologues of dTMP in SW620 WT and *MTHFD2*^*−/−*^ cells treated for 24 h with 1 μM TH9619 and TH9975 (cultured in RPMI-FBS), means ± s.d. (*n* = 3 for untreated and TH9619, *n* = 2 for TH9975); one-way ANOVA with Tukey’s multiple comparisons test. **l**, Relative enrichment of [2,3,3-^2^H]serine-derived M + 1 to M + 2 isotopologues of dTMP in SW620 WT and *MTHFD2*^*−/−*^ cells treated for 24 h with 1 μM TH9619 and TH9975 (cultured in RPMI-FBS), means ± s.d. (*n* = 3 for untreated and TH9619, *n* = 2 for TH9975); one-way ANOVA with Tukey’s multiple comparisons test for D + 1 isotopologue of dTMP. Source data

**Extended Data Fig. 2:**
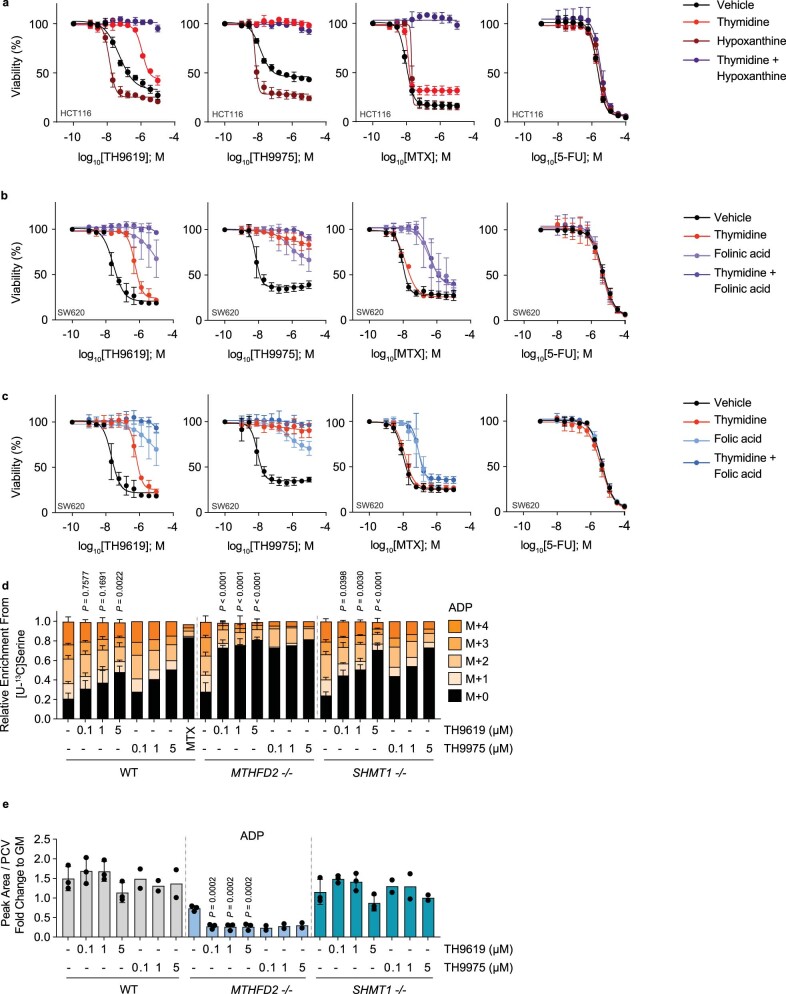
Thymidine and folinic acid or folic acid rescue TH9619 and TH9975 toxicity in SW620 WT cells. (**a**) Dose-response curves of HCT116 cells treated for 96 h with TH9619, TH9975, methotrexate (MTX) or 5-fluorouracil (5-FU) and 50 μM thymidine, 250 μM hypoxanthine or vehicle (cultured in RPMI-FBS), means ± SD (n = 3). (**b**) Dose-response curves of SW620 cells treated for 96 h with TH9619, TH9975, MTX or 5-FU and 50 μM thymidine, 1 μM folinic acid or vehicle (cultured in RPMI-FBS), means ± SD (n = 3). (**c**) Dose-response curves of SW620 cells treated for 96 h with TH9619, TH9975, MTX or 5-FU and 50 μM thymidine, 250 μM folic acid or vehicle (cultured in RPMI-FBS), means ± SD (n = 3). (**d**) [U-^13^C]serine- derived MID of ADP in SW620 WT, *MTHFD2*^*-/-*^ and *SHMT1*^*-/-*^ cells treated for 24 h with the indicated concentrations of TH9619 and TH9975 in μM or 50 nM MTX (cultured in RPMI-FBS), means ± SD (n = 3 for untreated and TH9619, n = 2 for TH9975, n = 1 for MTX); one-way ANOVA with Tukey’s multiple comparisons test for M + 0. *P*-values indicate comparisons to the untreated control of each cell line. (**e**) ADP levels normalized to packed cell volume (PCV) in SW620 WT, *MTHFD2*^*-/-*^ and *SHMT1*^*-/-*^ cells treated for 24 h with the indicated concentrations of TH9619 and TH9975 in μM (cultured in RPMI-FBS), mean ± SD (n = 3 for untreated and TH9619, n = 2 for TH9975); one-way ANOVA with Dunnett’s test for multiple comparisons. *P*-values indicate comparisons to the untreated control. Source data

SW620 and HCT116 colorectal cancer cells became partially resistant to TH9619 in the presence of thymidine, and fully resistant in the presence of thymidine plus hypoxanthine (Fig. 2a and Extended Data Fig. 2a) or with thymidine and folinic acid or folic acid (Extended Data Fig. 2b,c). Resistance to TH9975 toxicity was obtained by addition of thymidine (Fig. 2b and Extended Data Fig. 2a). Hypoxanthine moderately increased the sensitivity to TH9619 and TH9975 (Fig. 2a,b). This contrasts to all other compounds (Fig. 2c–f and Extended Data Fig. 2a,c). Instead, the effects of DS18561882 and SHIN1 were both partially reversed in the presence of hypoxanthine (Fig. 2c,d). Response also differed when treating with existing antifolates, as MTX resistance was induced by the combination of thymidine and hypoxanthine, but by neither alone, and no change was detected in response to 5-FU with any of the additives (Fig. 2e,f). This highlights that TH9619 has a unique mechanism of action that involves a primary toxicity due to thymidylate depletion, as well as a secondary toxicity due to purine deficiency.

To explore this further, we performed isotope tracing using [U-^13^C]serine and [2,3,3-^2^H]serine and measured newly synthesized purines (Fig. 2g) and thymidine (Fig. 2h), respectively, in response to TH9619 and TH9975 in SW620 WT, *MTHFD2*^*−/−*^ and *SHMT1*^*−/−*^ cells. Newly synthesized purines can be identified by the incorporation of [U-^13^C]serine-derived glycine and formate, which increases their mass (that is, M + 1, M + 2, M + 3, M + 4) due to incorporation of single or multiple ^13^C atoms from [U-^13^C]serine (Fig. 2g). To evaluate purine de novo synthesis rates during TH9619 and TH9975 treatment, we thus inspected the relative mass isotopomer distribution (MID) of these higher mass isoptopologues of ATP and ADP. SW620 WT cells showed a small reduction of ATP and ADP synthesis in response to TH9619 and TH9975 (Fig. 2i and Extended Data Fig. 2d). Untreated *SHMT1*^*−/−*^ cells were still able to synthesize purines in a similar capacity to SW620 WT cells and showed a modest reduction in isotope labelling compared to WT cells (Fig. 2i and Extended Data Fig. 2d) in response to TH9619 and TH9975 treatment. Owing to the reversibility of the cytosolic 1C cycle, untreated *MTHFD2*^*−/−*^ cells also retained the ability to synthesize purines in the absence of TH9619 or TH9975 treatments (Fig. 2i and Extended Data Fig. 2d). In stark contrast to WT and *SHMT1*^*−/−*^ cells, *MTHFD2*^*−/−*^ cells were, however, unable to synthesize ATP from the 1C cycle in response to TH9619 or TH9975 at all tested concentrations (Fig. 2i and Extended Data Fig. 2d). This loss of de novo purine synthesis was also reflected in a drop in total ATP and ADP levels (Fig. 2j and Extended Data Fig. 2e). This result is in line with our observation that TH9619 inhibits the DC domain of MTHFD1 as it shows that *MTHFD2*^*−/−*^ cells can neither use the mitochondrial nor the cytosolic route of 1C metabolism to synthesize purines from [U-^13^C]serine when treated with TH9619 or TH9975.

Deuterium (^2^H) labelling with [2,3,3-^2^H]serine allows the tracking of the origin of the methylene-THF that is used for thymidylate synthesis (Fig. 2h). The cytosolic or nuclear route produces thymidylate with two deuterium atoms (D + 2) and the mitochondrial route produces thymidylate with one deuterium atom (D + 1)^13,29,30^. SW620 WT cells showed a high mitochondrial flux through MTHFD2 indicated by high D + 1/D + 2 ratio (Fig. 2k). Compared to WT cells, *MTHFD2*^*−/−*^ cells showed (independent of treatment) a near complete lack of the dTMP+1 isotopologue (Fig. 2l). Treatment of WT cells with TH9619 or TH9975 resulted in a loss of dTMP+1, indicating a reversal of the 1C flux with CH_2_-THF for dTMP synthesis being mainly derived via SHMT1. These results further indicate that TH9619 and TH9975 inhibit the DC domain activity of MTHFD1 and thus prevent incorporation of formate produced by the mitochondria into dTMP.

The metabolic tracing experiments revealed an interesting and unexpected mechanism of TH9619 in altering 1C metabolism flux and subsequent toxicity to cancer cells. The primary mechanism of TH9619 toxicity in SW620 WT cells is through thymidylate depletion because TH9619 inhibits the MTHFD1(DC) domain activity and thereby prevents the conversion of 10-CHO-THF to 5,10-CH_2_-THF for thymidylate biosynthesis. At this stage, it remained enigmatic to us why thymidine induced resistance to TH9619 and TH9975, given that SW620 WT cells should still be able to compensate for thymidylate synthesis through cytosolic SHMT1. We therefore further investigated how TH9619 and TH9975 cause thymidylate depletion.

### Hypoxanthine potentiates TH9619 by folate trapping

**Fig. 3:**
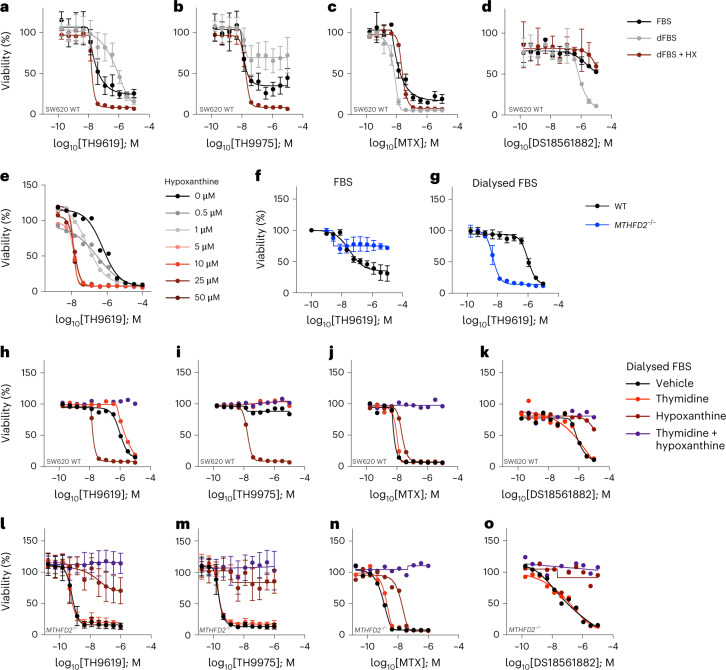
Hypoxanthine potentiates TH9619-induced thymidylate depletion. **a**–**d**, Cell viability dose–response curves of SW620 WT cells treated for 96 h with TH9619 (**a**), TH9975 (**b**), MTX (**c**) or DS18561882 (**d**), and cultured in RPMI-FBS, RPMI-dFBS or RPMI-dFBS supplemented with 50 μM hypoxanthine, means ± s.d. (*n* = 8 independent cell cultures for TH9619 and TH9975, *n* = 4 for MTX and DS18561882). **e**, Titration of hypoxanthine at indicated doses in SW620 WT cells treated for 96 h with TH9619 (cultured in RPMI-dFBS). Data are displayed as means (*n* = 2 independent cell cultures). **f**,**g**, Dose–response curves of SW620 WT and *MTHFD2*^*−/−*^ cells treated for 96 h with TH9619 and cultured in RPMI-FBS (**f**) or RPMI-dFBS (**g**), means ± s.d. (*n* = 3 independent cell cultures). **h**–**k**, Cell viability dose–response curves of SW620 WT cells treated for 96 h with TH9619 (**h**), TH9975 (**i**), MTX (**j**) or DS18561882 (**k**) and 50 μM thymidine, 50 μM hypoxanthine or vehicle (cultured in RPMI-dFBS), means (*n* = 2 independent cell cultures). **l**–**o**, Cell viability dose–response curves of SW620 *MTHFD2*^*−/−*^ cells treated for 96 h with TH9619 (**l**), TH9975 (**m**), MTX (**n**) or DS18561882 (**o**) and 50 μM thymidine, 50 μM hypoxanthine or vehicle (cultured in RPMI-dFBS), means ± s.d. (*n* = 4 independent cell cultures for TH9619 and TH9975, *n* = 2 for MTX and DS18561882). Source data

**Extended Data Fig. 3:**
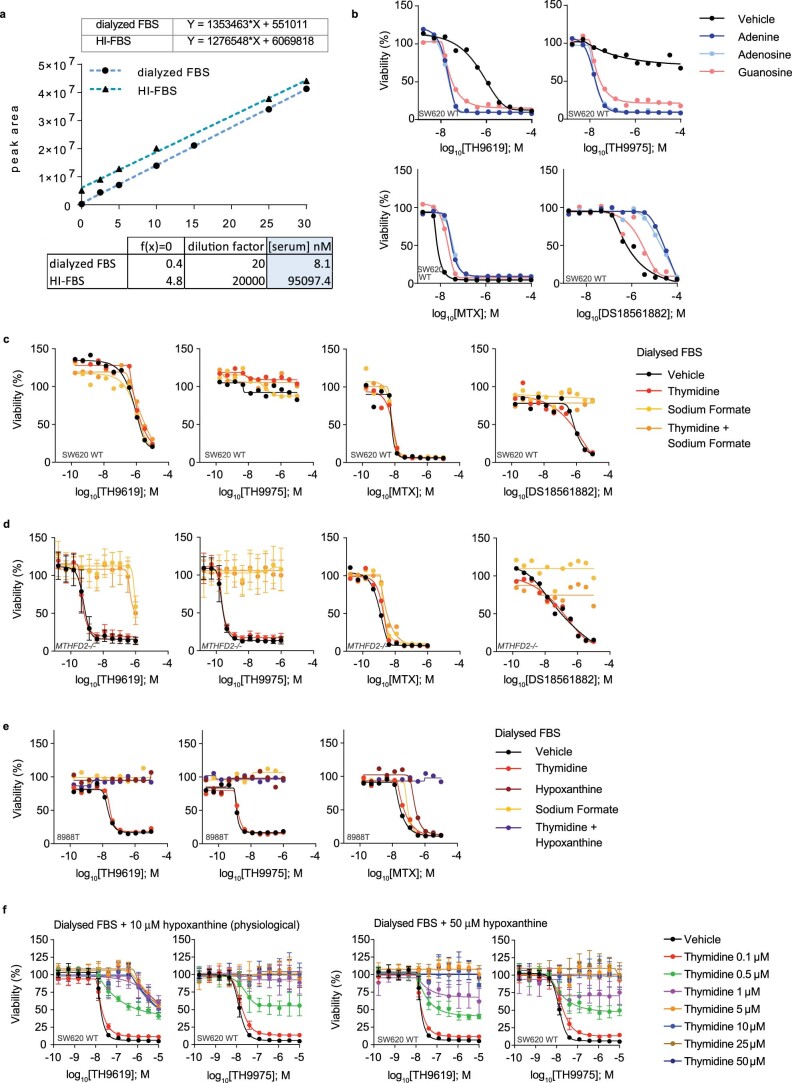
External supply of sodium formate rescues viability of cells with impaired mitochondrial 1C folate cycle. (**a**) HX concentration in HI-FBS and dialyzed FBS was determined by titration with HX standard of known concentration. Linear regression of the standard curve was used to calculate y-intercept and corresponding HX concentration in nM in pure HI-FBS or dialyzed FBS. (**b**) Dose-response curves of SW620 WT cells treated for 96 h with TH9619, TH9975, methotrexate (MTX) or DS18561882 and 50 μM adenine, 50 μM adenosine, 50 μM guanosine or vehicle (cultured in RPMI-dFBS), means (n = 2 independent cell cultures). (**c**) Dose-response curves of SW620 WT cells treated for 96 h with TH9619, TH9975, MTX or DS18561882 and 50 μM thymidine, 1 mM sodium formate or vehicle (cultured in RPMI-dFBS), means (n = 2 independent cell cultures). (**d**) Dose-response curves of SW620 *MTHFD2*^*-/-*^ cells treated as in (**c**). Means ± SD (n = 4 independent cell cultures for TH9619 and TH9975, n = 2 for MTX and DS18561882). (**e**) Cell viability dose-response curves of PA-TU-8988T cells treated for 96 h with TH9619, TH9975 or MTX and 50 μM thymidine, 50 μM hypoxanthine, 1 mM sodium formate or vehicle (cultured in DMEM-dFBS), means (n = 2 independent cell cultures). (**f**) Dose-response curves of SW620 WT cells treated for 96 h with TH9619 or TH9975 and increasing doses of thymidine. Cells were cultured in media containing dFBS and 10 or 50 µM hypoxanthine, means ± SD (n = 4). Source data

In early experiments, we noted that some cells were sensitized to TH9619 and TH9975 by the addition of hypoxanthine (Fig. 2a,b and Extended Data Fig. 2a). Until this point our experiments were performed in Roswell Park Memorial Institute (RPMI)-1640 culture media supplemented with normal, heat-inactivated foetal bovine serum (FBS). RPMI medium does not contain thymidine or purines, which can alter the sensitivity to our inhibitors, but normal FBS contains hypoxanthine. In our hands, normal FBS contains roughly 95 μM hypoxanthine (equating to 9.5 μM in RPMI + 10% FBS) and full sensitization to TH9619 occurs somewhere between 1 and 5 μM hypoxanthine (Fig. 3e and Extended Data Fig. 3a). Hence, to better understand the role of hypoxanthine, we performed subsequent experiments using media supplemented with dialysed FBS (dFBS) ± addition of 50 µM hypoxanthine.

When viability assays were repeated in RPMI supplemented with dFBS (RPMI-dFBS) lacking hypoxanthine, the toxicity in dose–response assays to TH9619 and TH9975 in SW620 WT cells was lost, and sensitivity was restored with supplementation of 50 μM hypoxanthine (Fig. 3a,b). In contrast, in RPMI-dFBS media SW620 cells were sensitized to MTX and DS18561882 (Fig. 3c,d), further suggesting a different mode of action of TH9619. Moreover, other purines including adenine, adenosine and guanosine increased the toxicity to TH9619 and TH9975, but partially rescued the toxicity of MTX and DS18561882 (Extended Data Fig. 3b). We also identified a key difference in sensitivity to TH9619 between WT and *MTHFD2*^*−/−*^ cells in FBS versus dFBS. As we reported previously^16^, compared to WT cells, *MTHFD2*^*−/−*^ cells display a decreased sensitivity to TH9619 in FBS (Fig. 3f). In stark contrast, TH9619 was several orders of magnitude more effective at reducing the viability of *MTHFD2*^*−/−*^ cells in dFBS compared to FBS (Fig. 3g). This suggested that the mechanism of TH9619 differs in cells with deficient or intact mitochondrial 1C metabolism.

We then performed metabolic rescue experiments with 50 μM thymidine, 50 μM hypoxanthine or 1 mM sodium formate using RPMI-dFBS in SW620 WT cells and in cells deficient in mitochondrial 1C metabolism: SW620 *MTHFD2*^*−/−*^ cells and MTHFD1L-defective PA-TU-8988T pancreatic cancer cells^13^ (Fig. 3h–o and Extended Data Fig. 3c–e). In SW620 WT cells, simultaneous supplementation of thymidine together with hypoxanthine caused resistance to TH9619 or TH9975 (Fig. 3h,i). In contrast, hypoxanthine mildly desensitized cells to MTX and provided resistance to DS18561882 (Fig. 3j,k). Moreover, the impact on cell viability in dFBS occurs downstream of mitochondrial formate production because sodium formate was unable to rescue the effect of TH9619 in WT cells (Extended Data Fig. 3c).

In contrast to WT cells, *MTHFD2*^*−/−*^ cells were resistant to TH9619 and TH9975 in the presence of hypoxanthine or sodium formate, but thymidine had no effect (Fig. 3l,m and Extended Data Fig. 3d). Similar results were obtained using PA-TU-8988T cells that are defective in MTHFD1L (ref. ^13^) (Extended Data Fig. 3e). Thus, in cells with defective mitochondrial 1C metabolism, the reduced viability is due to purine deficiency. This further supports our model that TH9619 causes toxicity by inhibiting the DC domain of MTHFD1.

**Fig. 4:**
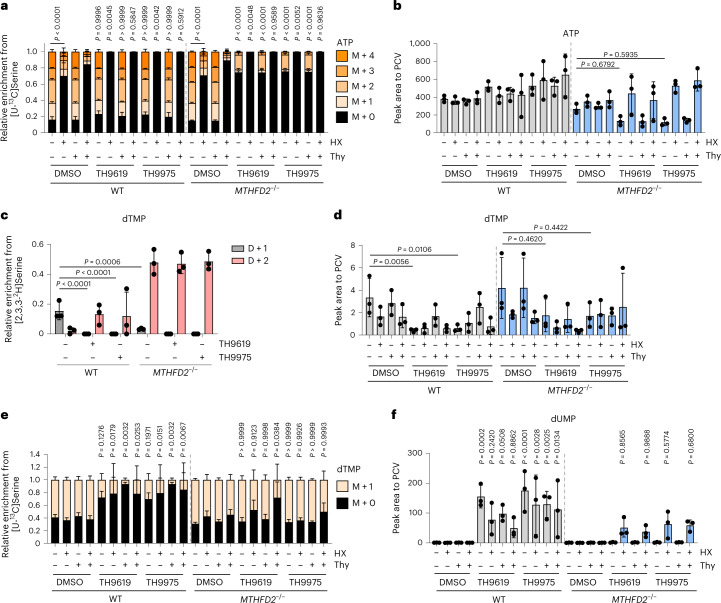
Metabolic tracing confirms potentiation of TH9619-induced thymidylate depletion by hypoxanthine. **a**, [U-^13^C]serine- derived MID of ATP in SW620 WT and *MTHFD2*^*−/−*^ cells treated for 24 h with 1 μM TH9619 or TH9975, 50 μM hypoxanthine and 50 μM thymidine (cultured in RPMI-dFBS), means ± s.d. (*n* = 3); one-way ANOVA with Šídák’s multiple comparisons test for M + 0. *P* values indicate comparisons to DMSO controls of each cell line. **b**, ATP levels normalized to PCV in SW620 WT and *MTHFD2*^*−/−*^ cells treated for 24 h with 1 μM TH9619 or TH9975, 50 μM hypoxanthine and 50 μM thymidine (cultured in RPMI-dFBS), means ± s.d. (*n* = 3); one-way ANOVA with Šídák’s multiple comparisons test. **c**, Relative enrichment of [2,3,3-^2^H]serine-derived M + 1 to M + 2 isotopologues of dTMP in SW620 WT and *MTHFD2*^*−/−*^ cells treated for 24 h with 1 μM TH9619 and TH9975 (cultured in RPMI-dFBS), means ± s.d. (*n* = 3); one-way ANOVA with Tukey’s multiple comparisons test. **d**, dTMP levels normalized to PCV in SW620 WT and *MTHFD2*^*−/−*^ cells treated for 24 h with 1 μM TH9619 or TH9975, 50 μM hypoxanthine and 50 μM thymidine (cultured in RPMI-dFBS), means ± s.d. (*n* = 3); one-way ANOVA with Šídák’s multiple comparisons test. **e**, [U-^13^C]serine-derived MID of dTMP in SW620 WT and *MTHFD2*^*−/−*^ cells treated for 24 h with 1 μM TH9619 or TH9975, 50 μM hypoxanthine and 50 μM thymidine (cultured in RPMI-dFBS), means ± s.d. (*n* = 3); one-way ANOVA with Šídák’s multiple comparisons test for M + 0. *P* values indicate comparisons to respective DMSO controls of each cell line. **f**, dUMP levels normalized to PCV in SW620 WT and *MTHFD2*^*−/−*^ cells treated for 24 h with 1 μM TH9619 or TH9975, 50 μM hypoxanthine and 50 μM thymidine (cultured in RPMI-dFBS), means ± s.d. (*n* = 3); one-way ANOVA with Šídák’s multiple comparisons test. *P* values indicate comparisons to respective DMSO controls of each cell line. Source data

**Extended Data Fig. 4:**
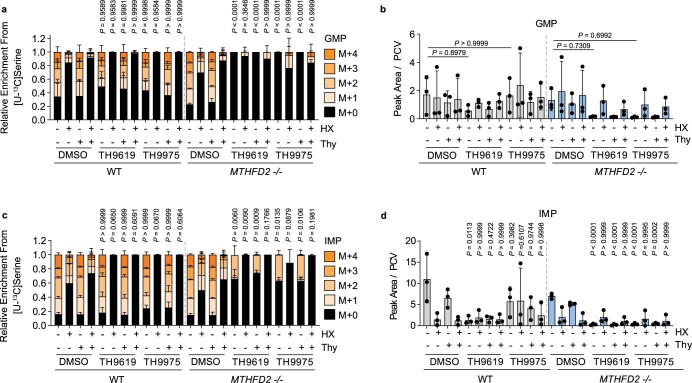
TH9619 and TH9975 impair GMP synthesis in SW620 *MTHFD2-/-* cells and reduce IMP levels in SW620 WT and *MTHFD2-/-* cells. (**a**–**d**) [U-^13^C]serine- derived MID of GMP (**a**) and IMP (**c**) and levels of GMP (**b**) and IMP (**d**) normalized to packed cell volume (PCV) in SW620 WT and *MTHFD2*^*-/-*^ cells treated for 24 h with 1 μM TH9619 or TH9975, 50 μM hypoxanthine and 50 μM thymidine (cultured in RPMI-dFBS), means ± SD (n = 3); one-way ANOVA with Šídák’s multiple comparisons test. For panels a and c, statistical analysis was performed for M + 0. *P*-values indicate comparisons to respective DMSO controls. Source data

This prompted us to investigate whether TH9619 and TH9975 effects on 1C-dependent nucleotide synthesis changed by culturing in RPMI-dFBS, thus we repeated [U-^13^C]serine and [2,3,3-^2^H]serine tracing in dFBS. In dFBS, de novo purine synthesis (ATP, guanosine monophosphate (GMP), inosine monophosphate (IMP)) from serine appeared mostly unaffected in SW620 WT cells following treatment with 1 μM TH9619 or TH9975 (Fig. 4a and Extended Data Fig. 4a–d). By contrast, serine-dependent de novo purine synthesis was severely impaired with TH9619 and TH9975 in *MTHFD2*^*−/−*^ cells (Fig. 4a and Extended Data Fig. 4a–d), which is reflected in lower total levels of ATP, GMP and IMP (Fig. 4b and Extended Data Fig. 4b,d). The addition of exogenous hypoxanthine caused a complete block in serine-derived de novo purine synthesis in both WT and *MTHFD2*^*−/−*^ cells (Fig. 4a and Extended Data Fig. 4a,c).

Similar to tracing experiments in FBS, in dFBS treatment with TH9619 or TH9975, or depletion of MTHFD2 prevented mitochondrial production of dTMP (D + 1) and reversed 1C flux to produce dTMP via the cytosol (D + 2) (Fig. 4c). *MTHFD2*^*−/−*^ cells treated with TH9619 or TH9975 displayed only a moderate reduction of total dTMP levels (Fig. 4d), while dTMP levels and de novo dTMP synthesis from serine in WT cells were strongly reduced (Fig. 4d,e). In line with dTMP synthesis inhibition, we observed a stark accumulation of dUMP, the precursor for dTMP synthesis in WT cells (Fig. 4f). This effect was much less pronounced in *MTHFD2*^*−/−*^ cells.

In summary, our data indicate that in cells with intact mitochondrial 1C metabolism, TH9619 and TH9975 cause thymidylate depletion and that this toxicity is dependent on hypoxanthine. By contrast, *MTHFD2*^*−/−*^ cells treated with TH9619 and TH9975 suffer from loss of purine synthesis and are resistant when cultured with hypoxanthine.

**Fig. 5:**
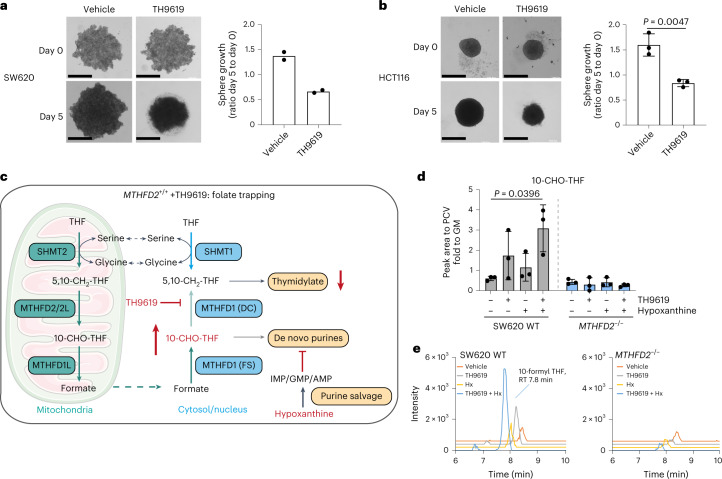
TH9619 and hypoxanthine causes 10-CHO-THF accumulation. **a**,**b**, HCT116 (**a**) and SW620 (**b**) cells were grown as spheroid cultures in three dimensions. Proliferation was measured as spheroid area from day 0 to 5 on 1 µM TH9619 or vehicle, means ± s.d. (*n* = 3 for HCT116 and *n* = 2 for SW620). Representative pictures shown for both cell lines. Scale bars, 500 μm. **c**, In the folate trapping model, hypoxanthine is converted to IMP, GMP or AMP by purine salvage, causing feedback inhibition on de novo purine synthesis. Simultaneously, TH9619 inhibits MTHFD1(DC), which together blocks consumption of 10-CHO-THF, creating a folate trap and preventing thymidylate synthesis due to exhaustion of THF. **d**, 10-CHO-THF levels normalized to PCV in SW620 WT and *MTHFD2*^*−/−*^ cells treated for 24 h with 1 μM TH9619 and 50 μM hypoxanthine (cultured in RPMI-dFBS), means ± s.d. (*n* = 3); one-way ANOVA with Tukey’s multiple comparisons test. **e**, LC–MS chromatograms for one representative experiment of **d**. RT, retention time. Source data

**Extended Data Fig. 5:**
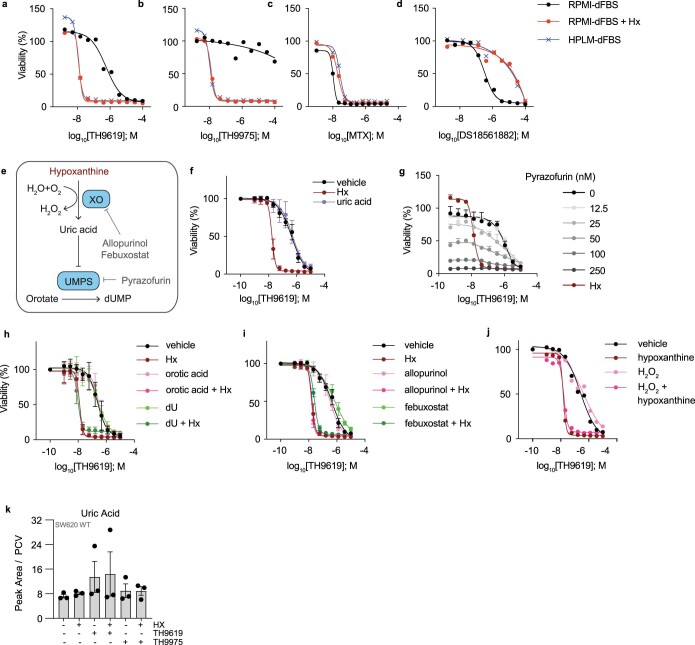
Inhibition of UMPS is not a major contributor to MTHFD1/2i toxicity. (**a**–**d**) Dose-response curves of SW620 cells treated for 96 h with TH9619, TH9975, methotrexate (MTX) or DS18561882 (cultured in RPMI-dFBS, RPMI-dFBS with 50 μM hypoxanthine or HPLM-dFBs), one representative experiment displayed as means (n = 2 independent cell cultures). (**e**) Hypothesis that hypoxanthine converts to uric acid to inhibit UMPS. (**f**) Dose-response curves of SW620 cells treated for 96 h with TH9619, 50 μM hypoxanthine, 350 μM uric acid or vehicle (cultured in RPMI-dFBS), means ± SD (n = 3). (**g**) Dose-response curves of SW620 cells treated for 96 h with TH9619, pyrazofurin or 50 μM hypoxanthine (cultured in RPMI-dFBS), means ± SD (n = 3 independent cell cultures). (**h**) Dose-response curves of SW620 cells treated for 96 h with TH9619, 50 μM hypoxanthine, 1 mM orotic acid, 500 μM deoxyuridine (dU) or vehicle (cultured in RPMI-dFBS), means ± SD (n = 3). (**i**) Dose-response curves of SW620 cells treated for 96 h with TH9619, 50 μM hypoxanthine (Hx), 10 μM allopurinol, 10 μM febuxostat or vehicles (cultured in RPMI-dFBS), means ± SD (n = 3). (**j**) Dose-response curves of SW620 cells treated for 96 h with TH9619 and 50 μM hypoxanthine, 100 μM H_2_O_2_ or vehicle (cultured in RPMI-dFBS), means (n = 2). (**k**) Uric acid levels normalized to PCV in SW620 WT cells treated for 24 h with 1 μM TH9619 or TH9975 and 50 μM hypoxanthine (cultured in RPMI-dFBS), means ± SD (n = 3). Source data

These findings also highlight the importance of cell culture media formulations in studies of 1C metabolism^31,32^. To ensure that these findings are clinically relevant, we compared drug responses in human plasma-like medium (HPLM)^32^ and Plasmax^31^ that have been developed to contain levels of nutrients comparable to human plasma, including 10 μM (HPLM) or 5 μM (Plasmax) hypoxanthine. SW620 WT cells were highly sensitive to TH9619 and TH9975 when cultured in HPLM, identical to RPMI-dFBS + 10 μM hypoxanthine (Extended Data Fig. 5a,b). In contrast, the presence of hypoxanthine in HPLM provided a slight resistance to MTX and several orders of magnitude lower sensitivity to DS18561882 (Extended Data Fig. 5c,d). Similarly, TH9619 reduced SW620 and HCT116 spheroid growth in physiological Plasmax medium (Fig. 5a,b). This supports that TH9619 is likely to be effective under human physiological conditions.

**Extended Data Fig. 6:**
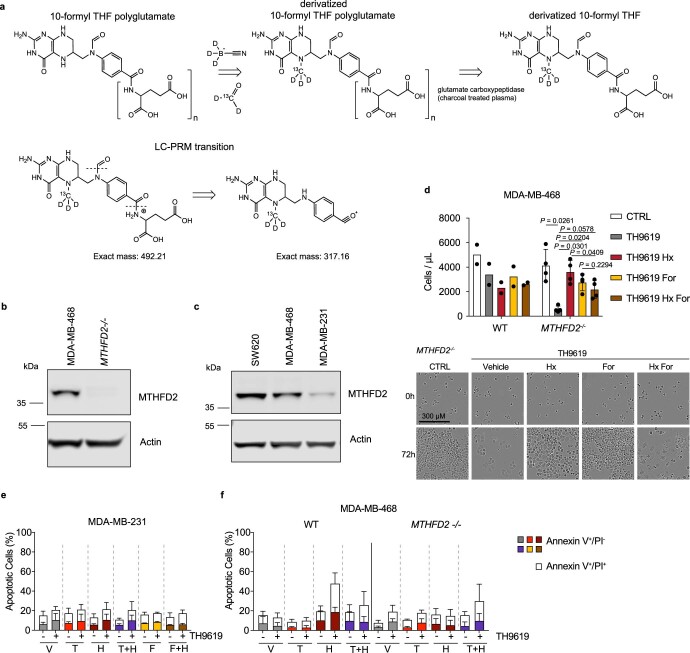
Breast cancer cells MDA-MB-468 can be sensitized to TH9619. (**a**) Top: Reductive methylation reaction described by Schittmayer *et al*.^42^ to stabilize the THF intermediates, followed by the cleavage of the glutamate tails by glutamate carboxypeptidase^43^. Bottom: Suggested mechanism for the tandem mass spectrometry fragmentation of 10-CHO-THF. (**b**, **c**) Protein expression of MTHFD2 was analyzed by Western Blot in MDA-MB-468 WT, MDA-MB-468 *MTHFD2*^*-/-*^, MDA-MB-231 and SW620 cells to validate MTHFD2 KO in MDA-MB-468 cells and different expression levels in WT cells. β-actin was used as loading control. Images are representative of 3 independent repeats. Signal intensity was quantified and normalized to loading control and global mean and is shown in Fig. 7i. (**d**) Absolute cell count was measured as cell number/uL by flow cytometry in MDA-MB-468 WT and MDA-MB-468 *MTHFD2*^-/-^ in response to 96 h treatment with 1 μM TH9619 and 50 μM hypoxanthine and 1 mM formate (cultured in RPMI-dFBS), means ± SD (n = 2 for WT and n = 4 for *MTHFD2*^*-/-*^); one-way ANOVA with Tukey’s multiple comparisons test was performed for *MTHFD2*^*-/-*^ cells. Representative images for Fig. 7k of MDA-MB-468 *MTHFD2*^-/-^ cell density and morphology are shown at 0 h and 72 h after treatment with 1 μM TH9619, 50 μM hypoxanthine and 1 mM formate (cultured in RPMI-dFBS). (**e**, **f**) Cell death analysis measured by AnnexinV/PI staining of MDA-MB-231, MDA-MB-468 WT and *MTHFD2*^*-/-*^ cells in response to 96 h treatment with 1 μM TH9619, 50 μM hypoxanthine (H), 50 μM thymidine (T) and 1 mM formate (F, cultured in RPMI-dFBS), means ± SD (n = 2-6). Source data

We proposed that the hypoxanthine-induced inhibition of de novo purine synthesis may be an important contributor to the mechanism of TH9619 and TH9975. In the purine salvage pathway, hypoxanthine is converted to IMP by hypoxanthine phosphoribosyltransferase 1 (HPRT1). IMP and its downstream products AMP and GMP are in turn inhibitors of phosphoribosyl pyrophosphate amidotransferase (PPAT), the first enzyme in the de novo purine synthesis pathway^33,34^. A reduced flow through de novo purine synthesis would lead to a reduced consumption of 10-CHO-THF because 10-CHO-THF is a cofactor for enzymes in the de novo purine synthesis pathway; glycinamide ribonucleotide transformylase and 5-amino-4-imidazolecarboxamide ribonucleotide transformylase (AICAR Tfase). In addition, if MTHFD1(DC) is inhibited by TH9619 or TH9975, this would prevent 10-CHO-THF consumption for both purine and thymidylate synthesis and block the re-synthesis of serine from glycine (Fig. 5c). Simultaneously, we have observed that formate continues to be produced from the mitochondria (Fig. 1f) and high formate concentrations will push the THF:10-CHO-THF equilibrium towards 10-CHO-THF. This scenario with a high synthesis and a small consumption of 10-CHO-THF could lead to a trapping of 10-CHO-THF and depletion of free THF (Fig. 5c). Measuring folate intermediates is inherently challenging, however, we managed to detect 10-CHO-THF by scaling up experiments, adapting previously published protocols and using a modified derivatization method (Extended Data Fig. 6a). Indeed, we observed an increase in 10-CHO-THF levels with TH9619, which was further increased on addition of hypoxanthine (Fig. 5d,e), indicating that 10-CHO-THF is accumulating. Since THF is a substrate for SHMT1, this trapping of THF as 10-CHO-THF would explain why SHMT1 cannot support dTMP synthesis on MTHFD1(DC) inhibition. Of note, there was no accumulation of 10-CHO-THF in *MTHFD2*^*−/−*^ cells in the absence of formate overflow (Fig. 5d,e). Our previous [U-^13^C]serine tracing experiments using exogenous, unlabelled hypoxanthine further confirm this 10-CHO-THF trapping model. If hypoxanthine fuels the cellular purine pool via the salvage pathway, and thereby prevents de novo synthesis from serine, we expect a loss of the ^13^C-labelled purine isotopologues coming from [U-^13^C]-serine tracer. Indeed, addition of hypoxanthine to WT cells strongly reduced the ^13^C-labelled fraction in ATP and on combined TH9619 or TH9975 treatment, the ^13^C-labelled fraction completely vanished (Fig. 4a). We also tested and could exclude a role for hypoxanthine in potentiating TH9619 response due to inhibition of uridine monophosphate synthase (Extended Data Fig. 5e–k and Supplementary Discussion)^32^.

### Mitochondrial formate release drives TH9619 trapping of 10-CHO-THF

**Fig. 6:**
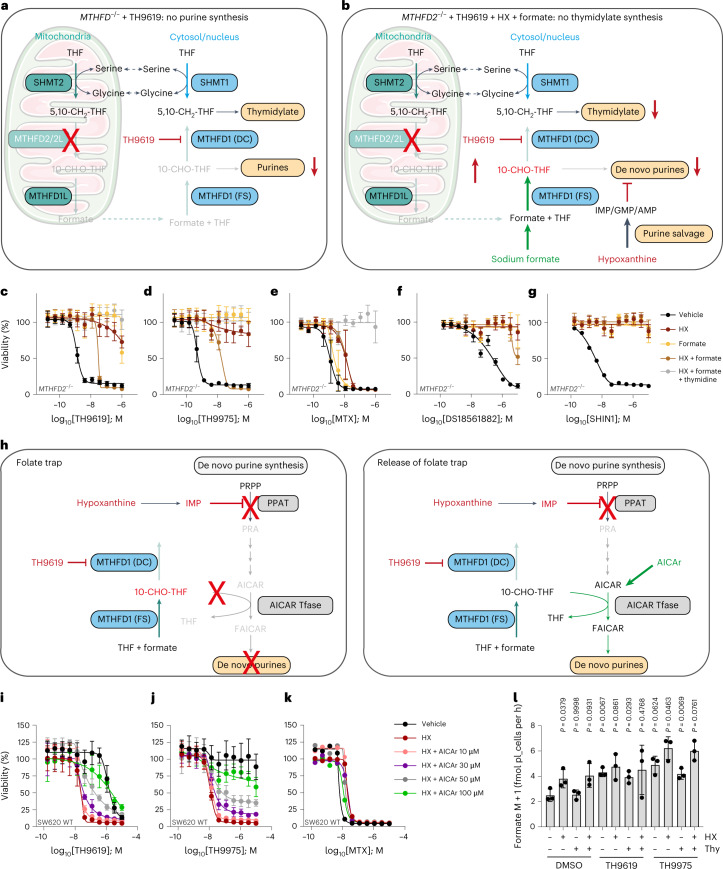
Formate release by mitochondrial 1C flux is required for TH9619-mediated trapping of 10-CHO-THF. **a**, TH9619 blocks purine synthesis in *MTHFD2*^*−/−*^ cells. **b**, Exogenous supply of sodium formate and hypoxanthine can induce the TH9619 folate trapping mechanism and block thymidylate synthesis in *MTHFD2*^*−/−*^ cells. **c**–**g**, Cell viability dose–response curves of SW620 *MTHFD2*^*−/−*^ cells treated for 96 h with TH9619 (**c**), TH9975 (**d**), MTX (**e**), DS18561882 (**f**) or SHIN1 (**g**) and 50 μM hypoxanthine, 1 mM sodium formate, 50 μM thymidine or vehicle (cultured in RPMI-dFBS), two representative experiments displayed as means ± s.d. (*n* = 4 independent cell cultures). **h**, Hypoxanthine and TH9619 cause folate trapping in WT cells. Treatment with AICAr bypasses the block in de novo purine synthesis and releases the folate trap by AICAR Tfase consumption of 10-CHO-THF. **i**–**k**, Cell viability dose–response curves of SW620 WT cells treated for 96 h with TH9619 (**i**), TH9975 (**j**) or MTX (**k**) and vehicle or increasing AICAr concentrations (cultured in RPMI-dFBS), two representative experiments are shown as means ± s.d. (*n* = 4 independent cell cultures for TH9619 and TH9965, *n* = 2 for MTX). **l**, [U-^13^C]serine-derived formate release rate of SW620 WT cells treated for 24 h with 1 μM TH9619 or TH9975, 50 μM hypoxanthine and 50 μM thymidine (cultured in RPMI-dFBS), means ± s.d. (*n* = 3); one-way ANOVA with Dunnett’s multiple comparisons test. *P* values indicate comparisons to the DMSO control. Source data

We next wanted to confirm that formate overflow was required to feed this folate trap and to cause thymidylate depletion. To investigate this, we tested whether a comparable toxic trapping of THF in the form of 10-CHO-THF could also be induced in *MTHFD2*^*−/−*^ cells that are unable to synthesize formate via the mitochondrial route (Fig. 6a). Supplementation of hypoxanthine or formate allowed *MTHFD2*^*−/−*^ cells to overcome purine deficiency by using hypoxanthine for purine synthesis or restoring formate, respectively (Fig. 3l,m and Extended Data Fig. 3d). If the trapping hypothesis is correct, the combination of TH9619, hypoxanthine and formate in *MTHFD2*^*−/−*^ would lead to trapping of 10-CHO-THF and thymidylate deficiency. Exogenous formate would mimic formate overflow and would be converted with free THF to 10-CHO-THF. 10-CHO-THF would now also accumulate in TH9619-treated *MTHFD2*^*−/−*^ cells because it is not being consumed for purine synthesis in the presence of hypoxanthine (Fig. 6b). The consequential depletion of free THF means that there is now also in *MTHFD2*^*−/−*^ cells no substrate available for SHMT1 and thymidylate production. In line with this hypothesis, a combination of hypoxanthine and formate induced TH9619 and TH9975 toxicity in *MTHFD2*^*−/−*^ cells (Fig. 6c,d). This toxicity was no longer due to purine deficiency, but instead caused by thymidylate deficiency and could thus be rescued by thymidine addition (Fig. 6b–d). The TH9619 and TH9975 toxicity in *MTHFD2*^*−/−*^ cells in the presence of hypoxanthine and formate in dFBS (TH9619 half-maximum effective concentration (EC_50_) of 11.6 nM, TH9975 EC_50_ = 10.7 nM) resembled that of SW620 WT cells when cultured in dFBS plus hypoxanthine (TH9619 EC_50_ = 16.5 nM, TH9975 EC_50_ = 17.4 nM) (Figs. 3a,b and 6c,d and Supplementary Table 1). This phenomenon may also exist with DS18561882 at much higher concentrations, but does not occur with MTX or SHIN1 (Fig. 6e–g). This suggests that inhibition of the DC domain of MTHFD1 and accumulation of 10-CHO-THF in a folate trap is key to the mechanism behind TH9619-dependent inhibition of thymidylate synthesis.

We were also interested to see whether a conversion of 10-CHO-THF to THF could save SW620 WT cells from toxicity caused by TH9619 or TH9975 plus hypoxanthine. We reasoned that because hypoxanthine leads to IMP-mediated inhibition of PPAT^33,34^, the synthesis of AICAR in de novo purine synthesis pathway would decrease (Fig. 6h). Since AICAR Tfase uses AICAR as a substrate and 10-CHO-THF as a cofactor, the 10-CHO-THF consumption decreases and results in further 10-CHO-THF trapping. Therefore, toxicity caused by TH9619 combined with hypoxanthine should be saved by addition of membrane permeable 5-amino-4-imidazolecarboxamide ribonucleoside (AICAr), which is intracellularly converted to AICAR by adenosine kinase. On hypoxanthine-mediated PPAT inhibition, such AICAr supplementation would thus increase the conversion of 10-CHO-THF to THF and the liberated THF can be used by SHMT1 to synthesize 5,10-CH_2_-THF that is required for dTMP synthesis. Indeed, AICAr dose-dependently reduced the toxicity of TH9619 or TH9975 in combination with hypoxanthine (Fig. 6i,j). The ability of combined hypoxanthine and formate treatment to trigger TH9619 or TH9975 toxicity via thymidylate deficiency in *MTHFD2*^*−/−*^ cells, along with AICAr rescue in SW620 WT cells strongly supports that 10-CHO-THF trapping causes toxicity due to thymidylate depletion. In comparison, MTX was unaffected by supplementation of AICAr (Fig. 6k), indicating that 10-CHO-THF trapping is a mechanism unique to this class of 1C inhibitors.

We have previously shown that formate overflow is a common trait in cancer cells that requires mitochondrial 1C metabolism^22–24^. We here propose that MTHFD2 supports formate overflow in cancer cells (as in Fig. 1f) rendering them sensitive to MTHFD1(DC) inhibition by TH9619. Indeed, we found that TH9619 and TH9975 increase formate overflow in SW620 WT cells and that this effect is most pronounced when either drug is combined with hypoxanthine, regardless of the presence of exogenous thymidine (Fig. 6l).

### dTMP depletion induces cell death and purine depletion is cytostatic

Thymidylate and purine biosynthesis are both essential for maintaining genomic integrity and supporting the high proliferative demands in cancer cells. As TH9619 exerts different effects on 1C metabolism flux in MTHFD2-expressing versus null cancers, we next explored whether these distinct metabolic changes also result in different biological effects in these cells and how hypoxanthine contributes to sensitization.

**Fig. 7:**
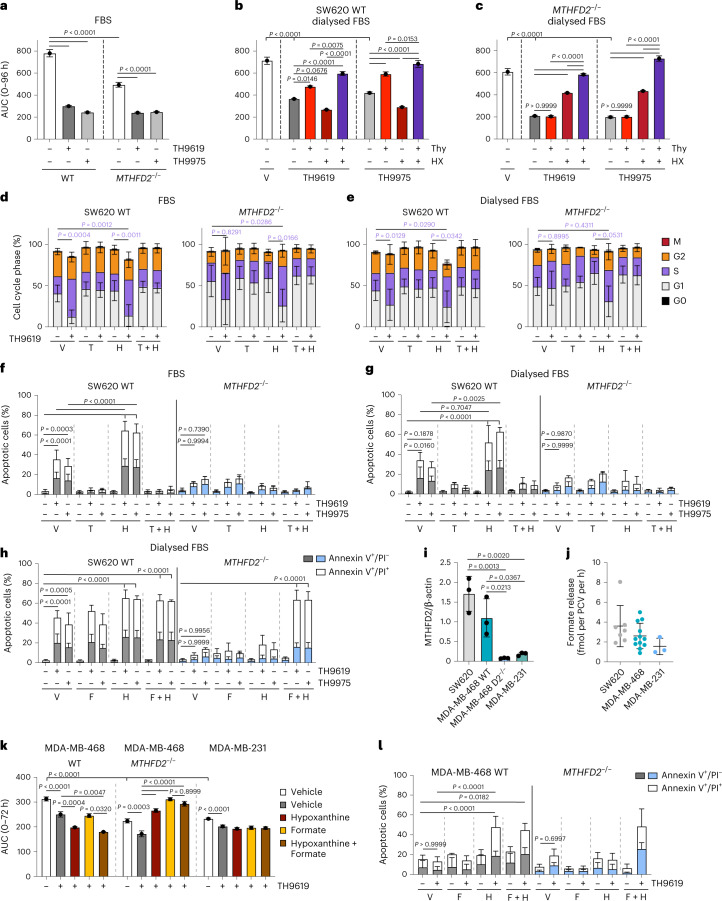
Depletion of dTMP induces cell death while inhibition of purine synthesis induces growth and cell cycle arrest. **a**–**c**, Proliferation of (**a**,**b**) SW620 WT and (**c**) *MTHFD2*^*−/−*^ cells measured as fold change of cell confluence after (**a**) 1 μM TH9619 or TH9975 (**b**,**c**) with or without 50 μM hypoxanthine and 50 μM thymidine (cultured in RPMI-FBS or RPMI-dFBS). AUC of proliferation curves is shown as means ± standard error ((**a**) *n* = 3 for WT, *n* = 4 for *MTHFD2*^*−/−*^ (**b**,**c**) *n* = 4); one-way ANOVA with (**a**) Šídák’s multiple comparisons test or (**b**,**c**) Tukey’s multiple comparisons test. **d**,**e**, Cell cycle analysis of SW620 WT and *MTHFD2*^*−/−*^ cells treated with TH9619, 50 μM thymidine (T), 250 μM hypoxanthine (H) or vehicle (cultured in (**d**) RPMI-FBS or (**e**) RPMI-dFBS), means ± s.d. (*n* = 3); one-way ANOVA with Tukey’s multiple comparisons test for S-phase. **f**–**h**, Cell death analysis by annexinV/PI staining of SW620 WT and *MTHFD2*^*−/−*^ cells after 96 h treatment with 1 μM TH9619 or TH9975 and (**f**,**g**) 50 μM hypoxanthine (H) and 50 μM thymidine (T) cultured in (**f**) RPMI-FBS or (**g**) RPMI-dFBS, or (**h**) treated with T and 1 mM formate (F) cultured in RPMI-dFBS, means ± s.d. (*n* = 3); one-way ANOVA analysis with Tukey’s multiple comparisons test for pooled early and late apoptotic populations. **i**, MTHFD2 protein levels in SW620, MDA-MB468 and MDA-MB-231 cells normalized to β-actin, means ± s.d. (*n* = 3); one-way ANOVA analysis with Tukey’s multiple comparisons test. **j**, Formate release rates of SW620, MDA-MB-468 and MDA-MB-231 cells, means ± s.d. (*n* = 7 for SW620, *n* = 12 for MDA-MB-468 and *n* = 3 for MDA-MB-231). **k**, Proliferation of MDA-MB-468 WT and *MTHFD2*^*−/−*^ cells and MDA-MB-231 cells measured as fold change of cell confluence in response to 1 μM TH9619 and 50 μM hypoxanthine and 1 mM sodium formate (cultured in RPMI-dFBS). AUC of curves is shown as means ± s.e. (*n* = 3 for MDA-MB-468 and *n* = 4 for MDA-MB-231); one-way ANOVA with Tukey’s multiple comparisons test. **l**, Cell death analysis of MDA-MB-468 WT and *MTHFD2*^*−/−*^ cells after 96 h treatment with 1 μM TH9619 and 50 μM hypoxanthine (H) and 1 mM formate (F) (cultured in RPMI-dFBS), means ± s.d. (*n* = 4); one-way ANOVA analysis with Tukey’s multiple comparisons test for pooled early and late apoptotic populations. Source data

We found that TH9619 and TH9975 impaired proliferation in SW620 WT and *MTHFD2*^*−/−*^ cells cultured in RPMI-FBS over a 96 h time course (Fig. 7a). Similarly, WT cells cultured in RPMI-dFBS exhibited reduced proliferation in response to TH9619 and TH9975 treatment, which was exacerbated by hypoxanthine while resistance was increased by the addition of thymidine (Fig. 7b). This contrasts with *MTHFD2*^*−/−*^ cells, which did not proliferate following TH9619 or TH9975 treatment and did not show resistance in response to thymidine supplementation, but in which proliferation could be restored with hypoxanthine supplementation (Fig. 7c).

The impaired proliferation in SW620 WT cells corresponded to cell cycle arrest in S-phase after 48 h of TH9619 treatment in cells cultured in RPMI-FBS with or without hypoxanthine supplementation (Fig. 7d). S-phase arrest also occurred in SW620 WT cells cultured in RPMI-dFBS, although this was slightly less pronounced (Fig. 7e). Cell cycle arrest could be rescued by the addition of thymidine to WT cells cultured in media with either FBS or dFBS (Fig. 7d,e). Similarly, *MTHFD2*^*−/−*^ cells also exhibited S-phase arrest in response to TH9619 when cultured in RPMI-FBS combined with hypoxanthine (Fig. 7d), but the S-phase arrest was less pronounced in RPMI-dFBS-hypoxanthine cultures (Fig. 7e).

In SW620 WT cells, cell cycle arrest was followed by induction of apoptosis on TH9619 and TH9975 treatment, which was exacerbated with hypoxanthine and rescued with thymidine irrespective of culturing with FBS or dFBS (Fig. 7f,g). This is in stark contrast to *MTHFD2*^*−/−*^ cells, which showed minimal induction of apoptosis following TH9619 or TH9975 treatment (Fig. 7f,g). This revealed that the reduced viability in resazurin assays following TH9619 treatment is caused by distinct biological mechanisms in WT and *MTHFD2*^*−/−*^ cells (Fig. 3h,i,l,m), depending on the drugs’ impact on 1C metabolism. TH9619 imposes a cytostatic effect on cancer cells with low MTHFD2 expression by restricting the pool of purines available for DNA synthesis. In comparison, thymidylate deficiency caused by TH9619 in SW620 WT cells with high MTHFD2 expression induces cell death, presumably due to induction of DNA damage by uracil misincorporation^5^. Finally, and in accordance with the folate trapping model, simultaneous addition of hypoxanthine and formate sensitized *MTHFD2*^*−/−*^ cells to TH9619 and TH9975 and resulted in a very strong induction of cell death (Fig. 7h).

To ensure that the described mechanism is also consistent across a broader panel of cell lines, we aimed to verify the effects of TH9619 treatment in breast cancer cell lines with different expression levels of MTHFD2. We confirmed different MTHFD2 protein expression in MDA-MB-231 (low MTHFD2 expression), MDA-MB-468 WT (high MTHFD2 expression) and MDA-MB-468 *MTHFD2*^*−/−*^ (no MTHFD2 expression) cells compared to SW620 WT cells (Fig. 7i and Extended Data Fig. 6b,c). Furthermore, MTHFD2 expression levels are reflected in different formate release rates (Fig. 7j). We found that TH9619 slowed proliferation in MDA-MB-468 WT, *MTHFD2*^*−/−*^ cells and MDA-MB-231 cells (Fig. 7k). In line with results in SW620 cells, proliferation of MDA-MB-468 WT cells was further reduced on combined treatment with hypoxanthine, while hypoxanthine addition restored proliferation in *MTHFD2*^*−/−*^ cells (Fig. 7b,c,k and Extended Data Fig. 6d). Of note, simultaneous supplementation of hypoxanthine and formate in TH9619-treated MDA-MB-468 *MTHFD2*^*−/−*^ cells caused an increase in cell size but not absolute cell numbers (Extended Data Fig. 6d), explaining the increase in confluency observed in the proliferation assay in these conditions (Fig. 7k). Comparable to SW620 cells, TH9619 toxicity in MDA-MB-468 WT cells was increased with hypoxanthine addition and unaltered by additional formate treatment (Fig. 7g,h,l). Moreover, similar to SW620 *MTHFD2*^*−/−*^ cells, TH9619 alone had minimal effects on apoptosis in MDA-MB-468 *MTHFD2*^*−/−*^ cells, while simultaneous addition of formate and hypoxanthine potently induced TH9619-dependent apoptosis (Fig. 7h,l).

Together, this indicates that TH9619 causes thymidylate deficiency in MTHFD2-expressing cancer cells by inhibiting MTHFD1(DC), leading to 10-CHO-THF trapping and depletion of THF. This mechanism relies on physiological levels of hypoxanthine to activate the purine salvage pathway and feedback inhibition of de novo purine synthesis. The drug effect in *MTHFD2*^*−/−*^ cells can be switched from cytostatic purine depletion to toxic inhibition of thymidylate biosynthesis by inducing 10-CHO-THF trapping on combinatorial hypoxanthine and formate supplementation. Given that formate levels are likely to be increased in certain tumours in vivo^22^, this may increase the pharmaceutical potential of TH9619. This will be important to ensure sensitivity of human tumours, given that physiological levels of purines may dampen the response to 1C drugs that work by primarily inhibiting purine synthesis^35^.

## Discussion

We previously developed inhibitors towards MTHFD2, such as TH9619, which turned out to be equipotent MTHFD1 and MTHFD2L inhibitors because of the considerable similarities of the active sites of these isoenzymes^16,36^. In this study, we reveal the complex biological mechanism of action behind TH9619 (and related TH9975) and show how it can be exploited to target both, MTHFD2-overexpressing cancers and cancers deficient in mitochondrial 1C metabolism.

We found that inhibition of the DC domain of MTHFD1 by TH9619 causes thymineless death specifically in MTHFD2-expressing cancer cells (Fig. 7f,g). This cell death induction occurs when MTHFD2-dependent formate is released from mitochondria and converted to 10-CHO-THF by the FS domain of MTHFD1. The 10-CHO-THF cannot be subsequently converted to 5,10-CH_2_-THF because the MTHFD1(DC) activity is inhibited by TH9619. This generates a ‘folate trap’ and 10-CHO-THF accumulates (Fig. 5c–e). At the same time, free THF is consumed and SHMT1 becomes starved of its THF substrate, which blocks thymidylate synthesis. This folate trap (10-CHO-THF accumulation) is dependent on physiological levels of hypoxanthine as hypoxanthine inhibition of de novo purine synthesis in conjunction with TH9619 inhibition of MTHFD1(DC), further prevents consumption of 10-CHO-THF. The reduced thymidine levels after hypoxanthine and TH9619 treatment suggest that SHMT1 activity and generation of 5,10-CH_2_-THF is impaired. We furthermore clearly demonstrate that treatment with TH9619 results in purine loss in *MTHFD2*^*−/−*^ cells (Fig. 2i,j) because 5,10-CH_2_-THF generated by SHMT1 cannot be converted to 10-CHO-THF and used for purine synthesis when TH9619 inhibits MTHFD1(DC) activity. Cytostatic effects of TH9619 in *MTHFD2*^*−/−*^ cells due purine depletion are fully rescued with either hypoxanthine or sodium formate but not with thymidine (Fig. 3l and Extended Data Fig. 3d) as SHMT1 upholds thymidine supply in *MTHFD2*^*−/−*^ cells. We were able to induce cytotoxic folate trapping in *MTHFD2*^*−/−*^ cells by combining TH9619 with sodium formate and hypoxanthine (Figs. 6c,d and 7h). This switched the mode of action from cytostatic purine deficiency to cytotoxic thymidylate deficiency and cell death, which could then be rescued with thymidine addition (Fig. 6c,d). These experiments neatly illustrate the folate trapping model and provide evidence for the mode of action of TH9619 and TH9975.

Previously, we reported that *MTHFD2*^*−/−*^, but not *MTHFD1*^*KD*^, cells were highly resistant to TH9619 treatment^16^, which suggested MTHFD2 rather than MTHFD1 was the drug target. This was not the case. Instead, we here show that mitochondrial MTHFD2 is not a drug target but that the observed resistance to TH9619 in *MTHFD2*^*−/−*^ cells is due to the lack of mitochondrial formate release. As a consequence, *MTHFD2*^*−/−*^ cells cannot feed the folate trap. As MTHFD1 is essential in many cancer cell lines^20^, the surviving MTHFD1 clones in our previous work may have had marginal expression of MTHFD1 (as detected by faint bands on western blots^16^). Resistance to TH9619 in *MTHFD1*^*KD*^ cells was not observed because they still express MTHFD2 and, as we now know, can thus generate a toxic folate trap. This is a lesson learned to exercise caution in CRISPR–Cas9 target validation, as in our case CRISPR–Cas9 deletion of the MTHFD2 protein (that is, node removal) resulted in an edgetic perturbation^37^ that changed the metabolic phenotype (that is, loss of mitochondrial formate release) of these cells. It was this change in phenotype, rather than the removal of the drug target, that made *MTHFD2*^*−/−*^ cells resistant to TH9619. Together this reveals that while MTHFD1(DC) is the drug target, it is formate overflow driven by MTHFD2 that sensitizes cells to thymidylate depletion and cell death upon TH9619 treatment. The lack of inhibition of mitochondrial MTHFD2 is probably due to the physicochemical properties of TH9619 and TH9975, which as other folate-like compounds require active transport over membranes by mitochondrial folate transporter. Instead, we found that it is nuclear MTHFD2 that is stabilized by TH9619. There may also be important roles for TH9619 interfering with nuclear functions of MTHFD2, but this is a topic for future studies.

Although TH9619 is highly effective in killing HL-60 cancer cell in vitro (EC_50_ = 11 nM), only a marginal anticancer effect was observed in vivo, even with validation of in vivo target engagement in the tumour^16^. This is because the 1C metabolite levels in mice do not model the physiological levels in humans. In mice, thymidine (roughly 100-fold) and folate (roughly 100-fold) levels are much higher while hypoxanthine is 100–1,000-fold lower than in humans^32,38^. A slightly better effect was observed by using low-folate diet, which assisted in reducing murine THF levels and hence reduced the ability of SHMT1 to use abundant THF as a substrate for thymidylate synthesis on TH9619 treatment. However, low-folate diet does not reduce high thymidine levels in mouse plasma^16^. Furthermore, with the gained mechanistic insights we now have, the poor efficacy of TH9619 in mice is probably also explained by low plasma levels of hypoxanthine in mice (roughly 0.02 µM)^32^, which is insufficient to inhibit the de novo purine pathway to support folate trapping. Therefore, mouse in vivo cancer models are probably unsuitable to predict TH9619 efficacy in humans. As such, a lack of appropriate animal models remains a limitation of this study.

**Extended Data Fig. 7:**
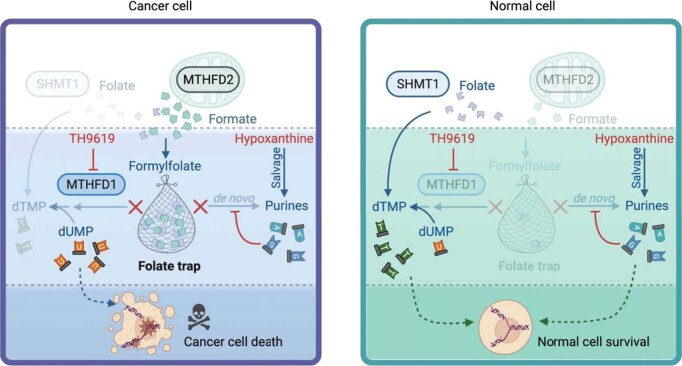
TH9619 creates a folate trap in MTHFD2-expressing cancer cells, but not normal cells. Schematic shows generation of a cancer-specific folate trap with TH9619 treatment. High MTHFD2 expression, which is common in cancer cells, causes formate overflow from the mitochondria. Human physiological levels of hypoxanthine activate the purine salvage pathway, causing feedback inhibition on *de novo* purine synthesis, and preventing consumption of 10-formyl-tetrahydrofolate (10-CHO-THF). Simultaneous inhibition of MTHFD1 with TH9619 also blocks consumption of 10-formyl-tetrahydrofolate for synthesis of thymidylate. This generates a folate trap and depletes cells of the THF substrate that can no longer be used to synthesize thymidylate via SHMT1. As a consequence, dUMP accumulates, and leads to toxic DNA damage and cancer cell death. Healthy cells do not express high levels of MTHFD2 and hence lack the formate overflow required to fuel the folate trap. Image created with BioRender.com.

Earlier, we reported that TH9619 and related compounds were highly toxic to cancer cells, while sparing non-malignant cells in vitro, demonstrating an impressive therapeutic index^16^. The reasoning was that MTHFD2 is an oncofetal protein, highly expressed during embryogenesis, largely silenced in adult cells and then re-expressed in cancer^6^. We have now discovered that the large therapeutic index is due to formate overflow, which we previously showed is a hallmark of cancer cells and requires mitochondrial 1C metabolism^22–24^. Without MTHFD2-driven formate release from mitochondria in cancer cells, the THF pool will not be depleted and 10-CHO-THF is not trapped (Extended Data Fig. 7). This is exemplified by the resistance of *MTHFD2*^*−/−*^ cells to TH9619 in the presence of hypoxanthine, resembling non-transformed cells with low levels of MTHFD2 expression. It is also important to note that in cell lysates TH9619 is also able to inhibit MTHFD2L. MTHFD2L is expressed in both, normal and cancer cells, albeit at lower levels than MTHFD2 (ref. ^39^). Owing to the structural similarity of both isozymes it may be challenging to develop a highly specific MTHFD2 inhibitor^36^. However, in the case of TH9619, the lack of effect on mitochondrial 1C metabolism means that side effects caused by targeting MTHFD2L are highly unlikely. Moreover, the folate trapping mechanism now provides an incentive for developing MTHFD1(DC)-specific inhibitors to treat MTHFD2-expressing cancers. Last, the cancer selectivity of TH9619 is also probably explained by increased target engagement with TH9619 in cancer cells as compared to non-transformed cells^16^. This is due to active uptake of folate-like compounds such as TH9619 in cancer cells through folate transporters to support the increased folate demand in cancer cells to drive biomass generation for rapid proliferation.

In conclusion, we describe a previously uncharacterized folate (specifically 10-CHO-THF) trapping mechanism, that is generated by inhibition of MTHFD1(DC) activity by TH9619 and related compounds. We demonstrate a mechanism of action distinct from other MTHFD1/2 inhibitors (DS18561882, ref. ^18^), traditional antifolates (MTX) or antimetabolites targeting TYMS (5-FU). We propose that cancer selectivity of TH9619 is related to the MTHFD2-dependent release of mitochondrial formate, which is a hallmark of cancer cells. Future work should be devoted to identifying the nuclear function of MTHFD2 through development of MTHFD1 and MTHFD2-selective chemical probes, identifying TH9619 transporters, response biomarkers and analysis of primary patients’ material under human physiological conditions to assist future clinical development. This study discovered that drugs targeting MTHFD1 can kill cancer cells that have upregulated MTHFD2, revealing cancer vulnerabilities that can be exploited for drug development.

## Methods

### Chemicals

Thymidine, hypoxanthine, sodium formate, adenine, adenosine, guanosine, deoxyuridine, H_2_O_2_ and pyrazofurin are from Sigma Aldrich. AICAr is from Chemtronica. Febuxostat is from Stratech Scientific Ltd. Allopurinol and Uric acid were from Merck Life Science UK.

### Cell culture

SW620 (CLL-227), HCT116 (CLL-247), MDA-MB-468 (HTB-132) and MDA-MB-231 (HTB-26) cells were from ATCC. PA-TU-8988T cells were from DSMZ (ACC162). SW620 and HCT116 WT cells were authenticated by short-tandem repeat analysis. SW620 *MTHFD2*^*−/−*^ and SW620 *SHTM1*^*−/−*^ cells were purchased from Synthego using single-guide RNA sequences targeting exon 2 (5′-CGCCAACCAGGAUCACACUC-3′ for *MTHFD2*^*−/−*^)^17^ or exon 4 (5′-UCAGGUGGCCCCCAUCCGGA-3′ for *SHMT1*^*−/−*^). MDA-MB-468 *MTHFD2*^*−/−*^ cells were generated as described previously^24^. All cell lines were cultured in RPMI GlutaMAX (Life Technologies), except for PA-TU-8988T cells that were maintained in DMEM GlutaMAX (Life Technologies). Unless otherwise specified, all media were supplemented with 10% heat-inactivated FBS (Life Technologies), 100 U ml^−1^ penicillin and 100 μg ml^−1^ streptomycin (Life Technologies). Cell lines were maintained at 37 °C in 5% CO_2_ humidified incubators, and routinely tested for mycoplasma using MycoAlertTM Kit (Lonza). HPLM was supplemented with 5% dFBS (Thermo Fisher Scientific). For experiments, 5 or 10% heat-inactivated or dFBS was used as indicated.

### Resazurin cell viability

Inhibitors were dissolved in dimethylsulfoxide (DMSO) at 10 mM and dispensed to final concentrations in 384-well plates (Corning) using Echo 550 Liquid handler (Labcyte) or D300e digital dispenser (Tecan). Cells were seeded at 1,000 (SW620) and 500 (HCT116) cells per well in 50 µl of complete medium. Plates were incubated in a humidified chamber for 96 h at 37 °C and 5% CO_2_. Cell viability was determined by adding 10 µg ml^−1^ resazurin (Sigma Aldrich) and measuring conversion to resorufin after 5 h. Fluorescence at 595 nm was measured with Hidex Sense reader and Hidex Sense Software v.1.0 (Hidex Oy) or SpectraMax iD5 reader (Molecular Devices). Half-maximal effective concentration (EC_50_) was calculated with logistic nonlinear regression in Prism v.9.3.1 (GraphPad).

### Mitochondrial membrane potential

SW620 cells were seeded at 5,000 per well in 96-well plates in complete medium 2–3 days before imaging. The next day, cells were treated with compounds for 48 h. Before imaging, cells were washed with PBS and incubated with 20 nM tetramethylrhodamine methyl ester (TMRM) (Molecular Probes) and 10 μM verapamil (Sigma Aldrich) for 30 min at 37 °C. Carbonyl cyanide p-trifluoromethoxyphenylhydrazone (FCCP, 20 μM, Sigma Aldrich) was used as a positive control. Cells were imaged live withSpark Cyto (Tecan) reader. TMRM fluorescence analysis in mitochondrial regions of interest was performed using CellProfiler.

### Mitochondrial isolation and CETSA

Here, 2.5 × 10^6^ SW620 cells were seeded on 150 mm dishes and grown for 4 days, followed by treatment for 3 h at 37 °C and 5% CO_2_ in a humidified incubator. Mitochondria were isolated using Qproteome Mitochondria Isolation Kit (Qiagen, catalogue no. 37612), and mitochondrial material from two fractionations were pooled before proceeding to CETSA. Briefly, cells were gathered and normalized to cell count. Cells were washed in 0.9% NaCl and lysed for 10 min in 1 ml lysis buffer with protease inhibitors. To isolate cytosolic protein from mitochondria and nuclei, cells were centrifuged at 1,000*g*, 4 °C for 10 min. Pellet was resuspended in 1 ml disruption buffer with protease inhibitors and passed ten times through a 21-gauge needle, followed by centrifugation at 1,000*g*, 4 °C for 10 min. The supernatant, containing mitochondria, was saved and mitochondria were re-extracted from the pellet by resuspending in 500 µl disruption buffer, passing through a needle and centrifuging at 1,000*g*, 4 °C for 10 min. Mitochondrial supernatants were then centrifuged at 6,000*g*, 4 °C for 10 min. Mitochondrial pellets were washed in 1 ml mitochondrial storage buffer and centrifuged at 6,000*g*, 4 °C for 20 min. For CETSA, mitochondria were resuspended in 360 µl 1× TBS (50 mM Tris-HCl, 150 mM NaCl, pH 7.6) with protease inhibitors (Roche) and divided into 30 µl aliquots. Mitochondria were heated in a Veriti Thermal Cycles (Applied Biosystems) for 3 min at indicated temperatures (temperature interval of 2.5 °C increases, from 42 to 62 °C) and cooled for 5 min at room temperature for precipitation of denatured proteins. Disruption of mitochondrial membrane was achieved by 4× freeze–thaw cycle in dry ice for 3 min and 37 °C for 3 min. Samples were cleared at 17,000*g*, 4 °C for 20 min. Supernatant was prepared for western blot analysis.

### DARTS

Target engagement was determined using DARTS assay as previously described^16,26,40^. SW620 cells in logarithmic growth phase were collected by trypsinization and lysed 10 min on ice in M-PER buffer (Thermo Fisher Scientific) with protease inhibitors (Roche). Lysates were centrifuged at 16,000*g*, 4 °C for 10 min. Protein concentration was determined by Bradford method with Pierce Coomassie Plus Assay Reagent (Thermo Fisher Scientific). Samples were diluted in 1× TN buffer (50 mM Tris-HCl pH 8.0, 50 mM NaCl) and treated as indicated for 30 min at room temperature. Treated cell lysates were aliquoted into 20 µg protein aliquots and digested with pronase (Roche) at the indicated pronase to total protein ratios for 30 min at room temperature. Reaction was stopped by 4× Laemmli sample buffer (BioRad) with 100 mM dithiothreitol and boiling at 95 °C for 5 min. Protein stabilization was analysed by western blot.

For target engagement in intact cells, SW620 cells were treated in culture with indicated compounds for 3 h before collection, lysis and pronase treatment performed as described above.

### Production of MTHFD1-Q100A

Complementary DNA encoding human MTHFD1-Q100A was ordered from Thermo Fisher Scientific and subcloned into pET22b using NdeI and SalI restriction enzymes and the bacterial expression vector pET22b-GFP-CPDSalI (kind gift from M. Bogyo and A. Shen; Addgene, 38257). Expression results in MTHFD1-Q100A C-terminally fused to cysteine protease domain (CPD) with C-terminal hexahistidin-tag^37^. MTHFD1-Q100A expression vector was transformed into *Escherichia coli* BL21 DE3 (Millipore, D48406). Bacterial culture was grown to an optical density (OD_600_) of 0.6 when expression was induced by 1 mM isopropyl-β-d-thiogalactoside (Sigma Aldrich, I6758). Bacteria were cultured at 16 °C for 20 h and centrifuged at 5,000*g* for 20 min. Bacteria were lysed using BugBuster (Millipore, D48832) with complete protease inhibitor cocktail (Roche, 11697498001), Benzonase nuclease (Millipore, D48913, 2.5 mU ml^−1^) and lysozyme (Sigma, L6876, 0.25 mg ml^−1^). After rotation for 30 min at room temperature, lysate was cleared at 16,000*g*. Lysate was loaded onto 5 ml HisTrap HP column (GE Healthcare) equilibrated with buffer A (50 mM Tris-HCl pH 7.8, 250 mM NaCl, 5% glycerol) and washed with buffer A with 10 mM imidazole. Bound proteins were eluted with 40–500 mM imidazole gradient in buffer A. Fractions were analysed using SDS–PAGE and Coomassie G-250 colloidal staining. Fractions containing MTHFD1 DCQ100A were pooled and dialysed against buffer B (50 mM Tris-HCl pH 8.0, 150 mM NaCl, 10% glycerol, 1 mM TCEP) over night and incubated with 300 µM phytic acid (InsP_6_) for 3 h at 5 °C to cleave CPD-tag. The His-tagged CPD protein was removed by passing over a 5 ml HisTrap column equilibrated with buffer B. The flow through fractions were collected and analysed using SDS–PAGE with Coomassie G-250 staining. Fractions with pure MTHFD1 DCQ100A were pooled and the concentration was determined using the Coomassie Plus Protein Kit (Thermo Scientific) using bovine serium albumin as a standard. Glycerol was added to 40% and protein was kept as aliquots at −20 °C.

### MTHFD1 and MTHFD1-Q100A inhibition assay

Inhibition assay of MTHFD1 and MTHFD1-Q100A mutant was performed as described previously^16,40^. Assay buffer consisted of 25 mM MOPS (pH 7.3), 0.5 mM TCEP, 400 µM NADP^+^ and 0.005% TritonX100. NADP^+^ was purchased from Sigma (N5755). Working solution of folitixorin (1.2% DMSO) was 60 µM in assay buffer. MTHFD1 WT and MTHFD1-Q100A mutant were diluted 0.5 nM in the assay buffer. Assay was performed as for MTHFD2 described in detail previously^40^, but using white Non-Binding Surface 384-well plates (Corning, 3824) and with 1 µl of 2.75 mM Menadione (Sigma, M5625) in 20% DMSO 10 min before reading. The final concentration in 5 µl was 30 µM folitixorin, 400 µM NADP^+^, 0.25 nM MTHFD1 WT or MTHFD1-Q100A.

### TYMS, SHMT1 and SHMT2 inhibition assay

Inhibition of TYMS, SHTM1 and SHMT2 was measured as previously described^40^.

### CRISPR-Select

We carried out CRISPR-Select analysis as described previously^27^. SW620 cells were nucleofected with CRISPR-Select cassette composed of: (1) Alt-R SpCas9 Nuclease V3 with target specific genomic RNA (synthetic crRNA–tracrRNA duplex; Integrated DNA Technologies), (2) a single-stranded oligodeoxynucleotide (ssODN) repair template containing MTHFD1-Q100A and (3) an identical ssODN repair template containing a synonymous MTHFD1-Q100Q internal normalization mutation (WT′) (both Ultramer DNA oligonucleotides; Integrated DNA Technologies). Nucleofection was performed in a Lonza 4D-Nucleofectorwith SF Cell Line 4D-Nucleofector X Kit S and CM-130 pulse program. On day 2 postnucleofection, cell population was split in two and cultured with and without 10 µM TH9619. Medium was changed every 3 days and cells were split to roughly 20% when 70–80% confluency was reached. On day 25, 100 ng genomic DNA was used as template to PCR amplify and sequence MTHFD1 genomic target site by amplicon Next Generation Sequencing using the MiSeq Reagent Kit v.2 (Illumina) and MiSeq instrument (Illumina) according to manufacturer´s instructions. Data were analysed by CRISPResso2 online tool using default settings (https://crispresso.pinellolab.partners.org/submission) to determine frequencies of MTHFD1-Q100A and MTHFD1-Q100Q alleles in cell populations.

### Stable isotope tracing

Experiments with [U-^13^C]-serine and [2,3,3-^2^H]-serine (Cambridge Isotope Laboratories) were performed in customized serine-, glycine- and glutamine-free RPMI-1640 (Cell Culture Technologies, Switzerland) with 0.4 mM glycine, 2 mM glutamine, 0.4 mM serine tracer and 10% FBS (or dFBS). Then 200,000 cells were seeded in 500 μl medium in 24-well plates in triplicates for each condition and in parallel triplicates for cell count and volume determination (to calculate the packed cell volume (PCV)). The day after, medium was replaced by 500 μl tracer medium with and without treatment. Three untreated wells per cell line were counted for start PCV. After 24 h of culture, one set of triplicate wells per condition were counted for end PCV. A parallel set of triplicate wells was used to collect medium for formate release rates and to preform metabolite extraction for liquid chromatography–mass spectrometry (LC–MS) analysis.

### Formate release rates

Formate extraction, derivatization and quantification in medium from stable isotope tracing experiments was performed as described previously^23^.

### LC–MS analysis of metabolite levels and mass isotopologue distribution

We carried out LC–MS analysis as described previously^23^. Cells in 24-well plates were washed with 500 μl cold PBS and extracted with 200 μl extraction solvent (MeOH:acetonitrile:H_2_O, 50:30:20) for 5 min at 4 °C under agitation. Solvent was centrifuged full speed, 10 min at 4 °C and supernatant was submitted for analysis. The LC–MS setup consisted in a Vanquish Flex (Thermo Scientific) LC system configured in binary gradient and coupled with a Q-Exactive Plus mass spectrometer (Thermo Scientific) and piloted by Xcalibur software (v.4.4.16.14, Thermo Scientific). The column was a SeQuant ZIC-pHILIC (Merck) (2.1 × 150 mm, 5 µm) with a guard column (2.1 × 20 mm) and was heated at 45 °C in the column oven. Solvent A is 20 mM ammonium carbonate pH 9.2 with 5 µM of medronic acid. Solvent B is pure acetonitrile. Samples were eluted by linear gradient from 80% B to 20% B in 15 min at constant flow rate of 200 µl min^−1^. MS acquisition was performed with a polarity switch between positive and negative electrospray ionization modes. Full MS spectra were acquired from 75 to 1,000 *m*/*z* at 70,000 resolution (at 200 *m*/*z*) with an automatic gain control set to 1 × 10^6^ charges and a maximum ion trap fill time of 250 ms. Data were analysed with TraceFinder (v.4.1, Thermo Scientific) and Skyline software (v.21.2). Data normalization and natural isotope subtraction were performed using in house scripts as previously reported^23^.

### Analysis of dTMP deuterium labelling

Data were acquired with the above described LC–MS system in negative polarity using targeted selected ion monitoring (tSIM) method. Ions were filtered by the quadrupole with an isolation window of 8 Da centred on 323.061 *m*/*z* and accumulated in C-trap for a maximum of 250 ms and a maximum of 5 × 10^5^ charges. Ions were analysed by the orbitrap at a resolution of 140,000 (at 200 *m*/*z*). Data were analysed with TraceFinder (v.4.1) using a 30 ppm mass tolerance for data processing. Natural isotope subtraction using in house scripts as described previously^23^ was used to correct deuterium labelling for ^13^C natural isotope abundance.

### Quantification of hypoxanthine in FBS

Heat-inactivated FBS and dFBS were precipitated by a tenfold dilution in the extraction buffer (MeOH:acetonitrile:H_2_O, 50:30:20). Samples were further diluted 1:1 by hypoxanthine standard solution to yield seven calibration points from 0 to 30 nM of added hypoxanthine. Endogenous concentration of the hypoxanthine was calculated as the absolute value of the *x* intercept of the linear regression curve. FBS samples were diluted 1,000 times more than dFBS to match concentration range. Data were acquired with the above described LC–MS system but using parallel reaction monitoring acquisition. Hypoxanthine protonated cation was selected at unit resolution by the quadrupole, fragmented with a normalized collision energy of 40. Maximum ion accumulation time was set to 220 ms and resulting fragment ions were analysed by the orbitrap at a resolution of 70,000 (at 200 *m*/*z*). The tandem MS (MS/MS) signal of the hypoxanthine’s fragment ions 119.0353, 110.0350, 94.0400, 82.0400, 67.0292 and 55.0294 *m*/*z* was extracted and summed with Skyline software (v.21.2)^41^.

### Quantification of 10-formyl THF

Sample preparation was adapted from Schittmayer et al.^42^ and Chen et al.^43^ Here, 11 × 10^6^ SW620 WT and *MTHFD2*^*−/−*^ cells were seeded in 10 ml culture medium in 75 cm^2^ flasks. 24 h after seeding, medium was exchanged to 10 ml of RPMI-1640 with 10% dFBS and cells were treated as indicated for 24 h. Then 15–30 × 10^6^ cells were collected by trypsination and pellets were washed with PBS. Solution in PBS was counted to determine cell count and size for PCV normalisation. Then 500 μl of ice-cold derivatization buffer (80% methanol, 30 mM NaB_2_H_3_CN, 0.2% ^13^CO_2_H_2_ and 0.1% acetic acid) was added to cell pellets and incubated on ice for 60 min. After centrifugation, supernatant was collected and evaporated in vacuum centrifuge. Samples were suspended in 450 µl aqueous buffer (50 mM K_2_HPO_4_, 0.5% 2-mercaptoethanol pH 7.0), mixed with 25 µl of charcoal purified rat serum and incubated for 2 h at 37 °C to cleave poly-glutamate tails. Samples were acidified with 3.5 µl of formic acid and purified using Bond Elute-PH SPE columns (Agilent). Columns were conditioned with 1 ml of MeOH and 1 ml of 25 mM of ammonium acetate pH 4.0. Samples were loaded onto the columns, washed with 1 ml of 25 mM ammonium acetate pH 4 and eluted in 400 µl elution buffer (50% methanol, 0.5% 2-mercaptoethanol, 49.5% 25 mM ammonium acetate at pH 7.0). Eluates were evaporated and resuspended in 50 µl of 80% MeOH with 0.5% acetic acid for analysis by LC–MS. Data were acquired with the above described LC–MS system in parallel reaction monitoring acquisition mode with positive ionization. The derivatized precursor ion of 10-formyl THF (492.211 *m*/*z*) was selected by the quadrupole at unit resolution and fragmented at a normalized collision energy of 20. The resulting fragment ions were accumulated for up to 1,000 ms, with a maximum number 1 × 10^6^ charges and analysed by the orbitrap at a resolution of 140,000 (at 200 *m*/*z*). The MS/MS signal fragment-ion 317.1629 *m*/*z* was used for quantification as described by Schittmayer et al.^42^. The absence of interference with 5-formyl THF was controlled using pure standard solutions for 10-formyl-THF and 5-formyl THF. MS data were processed with Skyline software (v.22.2) with a 5 ppm mass error tolerance on the centroid signal.

### Proliferation

Cells were seeded at low density in 96-well plates in 4–8 technical replicates per condition. Cell proliferation was determined as cell confluence using the IncuCyte Live-Cell Analysis system and IncuCyte ZOOM 2021C and 2018A software (Essen Bioscience). Area under curve (AUC) of cell confluence curves from 0 to 96 h were calculated in GraphPad v.9 and displayed as means ± s.e.

### Cell cycle analysis by flow cytometry

SW620 cells were cultured for 48 h in RPMI containing 10% FBS or dFBS and treated as indicated. Cells were collected, washed and resuspended in PBS. Cells were resuspended in fixation/permeabilization buffer and washed in permeabilization buffer (Thermo Fisher Scientific). Cells were stained with Ki-67 eFluor 506 (1:20, BioLegend) and p-Histone H3 AlexaFluor488 (1:50, BioLegend) or isotype controls in permeabilization buffer at 4 °C in the dark. Cells were washed twice in permeabilization buffer then resuspended in 100 μl PBS containing 10 μg ml^−1^ Hoechst 33342 (Life Technologies) and 100 μg ml^−1^ RNaseA^5^ (Thermo Scientific) and incubated for 15 min at room temperature in the dark. Samples were analysed on a BD LSRII with BD FACSDiva software (BD Biosciences) and data analysed in FlowJo (v.10.7.2, BD).

### Cell death measurement using annexinV-PI staining

Cells were seeded at 100,000 cells per well in 1 ml in 12-well plates and treated the subsequent day as indicated. After 96 h, samples were collected and stained as previously^24^. Measurement was done at NovoCyte Quanteon (Agilent) with NovoExpress software (Agilent, v.1.5.0). Analysis was conducted in FlowJo (v.10.6.2, BD).

### Sphere growth assay

Cells were cultured in Ultra Low Attachment Flasks, collected in a 50 ml Falcon and centrifuged at 300*g* for 5 min. Pellet was resuspended in 1 ml of TripLE and incubated 5 min at 37 °C. Culture medium was added to deactivate TripLE (Thermo Fisher). Cell suspension was centrifuged 5 min at 300*g*, pellet was resuspended in 1 ml of Plasmax (custom made) and counted with CEDEX XS (INNOVATIS). Here 5,000 cells were seeded in each well of a 96-well, cell repellent-treated, U-shaped-bottom microplate (Greiner). The plate was centrifuged at 300*g* for 5 min and incubated for 2 days at 37 °C to allow sphere formation. Spheres were treated with 1 μM TH9619 and imaged on Cytation5 (Agilent) on day 0 and day 5 after treatment. Sphere size and area was measured using binary pictures and particle analysis in ImageJ.

### Western blot

Cell pellets were incubated for 30 min on ice in lysis buffer (150 mM NaCl, 1 mM EDTA, 50 mM Tris-HCl, 1% NP-40) and sonicated. Lysates were centrifuged at 13,000*g*, 10 min 4 °C. Supernatant was collected and stored at −80 °C. Protein concentration was determined by Bradford assay. 15–30 μg of total protein were loaded on RunBlue 4–12% Bis-Tris gels (Westburg) using 4× NuPage LDS Sample buffer (Thermo Fisher Scientific) with 10 mM DTT (Sigma Aldrich) and blotted onto nitrocellulose membrane according to standard protocols. Membranes were blocked with 5% milk-powder in tris-buffered saline with Tween and incubated with primary antibodies over night at 4 °C. Incubation with secondary antibody was performed light-protected for 2 h at room temperature. Detection was done with Odyssey CLx Infrared Imaging System (LI-COR). ImageStudioLite v.5.2 (LI-COR) was used for analysis.

### Antibodies

Antibodies used for western blotting were: rabbit anti-MTHFD1 (Thermo Fisher Scientific, PA5-42825; 1:1,000), rabbit anti-MTHFD1 (Atlas Antibodies, HPA-000704; 1:500), rabbit anti-MTHFD1L (Proteintech, 16113-1-AP; 1:1,000), rabbit anti-MTHFD2 (Cell Signaling Technology, 41377, D8W9U; 1:1,000), mouse anti-MTHFD2 (Abcam, ab56772; 1:500), rabbit anti-SHMT1 (Sigma Aldrich, HPA023314; 1:1,000), rabbit anti-SHMT2 (Sigma Aldrich, HPA020549; 1:1,000), rabbit anti-HSP60 (Abcam, ab46798; 1:1,000), rabbit anti-Vinculin (Cell Signaling Technology, 4650; 1:1,000), rabbit anti-Lamin A/C (Cell Signaling Technology, 2032; 1:1,000), mouse anti-α-Tubulin (Abcam, ab7291; 1:20,000), mouse anti-ATP synthase beta (Invitrogen, A21351; 1:1,000), rabbit anti-TOM20 (Proteintech, 11802-1-AP-150; 1:1,000), mouse anti-β-actin (Cell Signaling Technology, 3700, clone 8H10D10; 1:3,000) and mouse anti-SOD1 (Santa Cruz Biotechnology, sc-17767; 1:1,000). Secondary antibodies were: IRDye 680RD Goat Anti-Mouse IgG (H+L) (Li-Cor Biosciences, 926-68070; 1:10,000), IRDye 800CW Donkey Anti-Mouse IgG (H+L) (Li-Cor Biosciences, 926-32212; 1:5,000), IRDye 800CW Donkey Anti-Rabbit IgG (H+L) (Li-Cor Biosciences, 926-32213; 1:5,000 or 1:10,000), Peroxidase AffiniPure Donkey Anti-Rabbit IgG (Jackson ImmunoResearch, 711-035-152; 1:5,000) and Peroxidase AffiniPure Donkey Anti-Mouse IgG (Jackson ImmunoResearch, 715-035-150; 1:5,000).

### Chemical synthesis of TH9975

Please see the Supplementary Information for a detailed protocol.

### Quantification and statistical analysis

All data were plotted and statistical analysis was carried out in Prism v.9.3.1 (GraphPad Software). Data are plotted as means ± standard deviation (s.d.) or means ± standard error (AUC plots) as indicated in the figure legends. Standard deviation is indicated for all cases where *n* ≥ 3. The number of replicates (*n*) corresponds to the number of independent experiments or independent cell cultures and is indicated in the figure legends.

### Reporting summary

Further information on research design is available in the Nature Portfolio Reporting Summary linked to this article.

## Supplementary information

Supplementary Information Supplementary Methods, Discussion and Fig. 1.

Reporting Summary

Supplementary Table EC_50_ table.

Supplementary Data ChemDraw files of TH9619 and TH9975.

## Data Availability

All data generated or analysed during the present study, including source data, can be found in the article, Extended Data or Supplementary Information. Source data are provided with this paper.

## Source data

Source Data Fig. 1 Statistical source data.
Source Data Fig. 1 Unprocessed western blots.
Source Data Fig. 2 Statistical source data.
Source Data Fig. 3 Statistical source data.
Source Data Fig. 4 Statistical source data.
Source Data Fig. 5 Statistical source data.
Source Data Fig. 6 Statistical source data.
Source Data Fig. 7 Statistical source data.
Source Data Extended Data Fig. 1 Statistical source data.
Source Data Extended Data Fig. 1 Unprocessed western blots.
Source Data Extended Data Fig. 2 Statistical source data.
Source Data Extended Data Fig. 3 Statistical source data.
Source Data Extended Data Fig. 4 Statistical source data.
Source Data Extended Data Fig. 5 Statistical source data.
Source Data Extended Data Fig. 6 Statistical source data.
Source Data Extended Data Fig. 6 Unprocessed western blots.
