# Supplementary material for: Formate overflow drives toxic folate trapping in MTHFD1 inhibited cancer cells
Source: Nat Metab. 2023 Apr 3;5(4):642–59. doi: 10.1038/s42255-023-00771-5 (PMC10132981; doi:10.1038/s42255-023-00771-5)
Supplement: Supplementary file 1 — Supplementary Methods, Discussion and Fig. 1. [file 42255_2023_771_MOESM1_ESM.pdf]

nature metabolism

Article

https://doi.org/10.1038/s42255-023-00771-5

Formate overflow drives toxic folate trapping in MTHFD1 inhibited cancer cells

In the format provided by the authors and unedited

Supplementary Information

Formate overflow drives toxic folate trapping in MTHFD1 inhibited cancer cells

Green et al.

- Supplementary Methods
- Supplementary Discussion
- Supplementary Figure 1

Supplementary Methods

Chemical Synthesis of TH9975

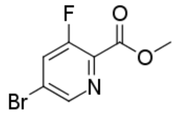

methyl 5-bromo-3-fluoropyridine-2-carboxylate

5-bromo-3-fluoropyridine-2-carboxylic acid (22 g, 100 mmol), MeOH (100 mL) and H_2SO_4 (9.8 g, 100 mmol) were refluxed for 4 h. The reaction was allowed to cool to RT and the solvent was reduced in a rotavapor. The mixture was diluted with water. $NaHCO_3$ (aq) was added until pH ~7. The mixture was extracted with EtOAc (4x) and combined organic phases were dried over Na_2SO_4 and removed in a rotavapor. Gave methyl 5-bromo-3-fluoropyridine-2-carboxylate (21 g, 91%). LCMS $[M+H]^+$ *m/z* 234, 236; ^1H NMR (400 MHz, DMSO-d_6) δ ppm 3.89 (s, 3H), 8.42 (dd, 1H), 8.70 (t, 1H).

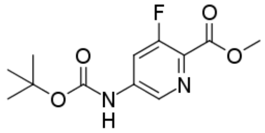

methyl 5-(tert-butoxycarbonylamino)-3-fluoro-pyridine-2-carboxylate

Methyl 5-bromo-3-fluoropyridine-2-carboxylate (2.4 g, 10 mmol), tert-butyl carbamate (1.4 g, 12 mmol), Cs_2CO_3 (3.9 g, 12 mmol) and dry dioxane (50 mL) was flashed six times with nitrogen. $Pd(OAc)_2$ (110 mg, 0.50 mmol) and X-Phos (480 mg, 1.0 mmol) were added and the mixture was flashed six times with nitrogen. The sealed reaction was heated at 90°C for 21 h. Brine (50 mL) and water (50 mL) were added and the mixture was extracted with EtOAc. The organic solvent was removed in a rotavapor and the product was purified by flash chromatograpghy (EtOAc/iso-hexane 25/75 to 50/50 as eluent). Gave methyl 5-{[(tert-butoxy)carbonyl]amino}-3-fluoropyridine-2-carboxylate (2.1 g, 76%). LCMS $[M+H]^+$ *m/z* 271; ^1H NMR (400 MHz, DMSO-d_6) δ ppm 1.49 (s, 9H), 3.85 (s, 3H), 7.92 (dd, 1H), 8.50 (t, 1H), 10.28 (br s, 1H).

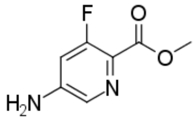

methyl 5-amino-3-fluoro-pyridine-2-carboxylate

TFA (2 mL) was added to a solution of methyl 5-(tert-butoxycarbonylamino)-3-fluoro-pyridine-2-carboxylate (190 mg, 0.70 mmol) in DCM (2 mL). The reaction was stirred at RT for 2 h and solvent was removed in vacuo. EtOAc (10 mL) was added to the crude material and the mixture was washed with sat. Na_2CO_3 (2x3 mL). The organic phase was dried over Na_2SO_4

and removed in a rotavapor. Gave methyl 5-amino-3-fluoro-pyridine-2-carboxylate (110 mg, 92%). LCMS $[M+H]^+$ *m/z* 171; ^1H NMR (400 MHz, DMSO-d_6) δ ppm 3.76 (s, 3 H) 6.54 (s, 2 H) 6.65 - 6.73 (m, 1 H) 7.81 - 7.86 (m, 1 H).

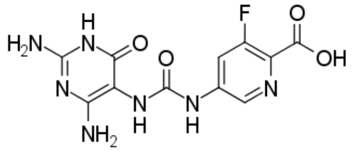

5-[(2,4-diamino-6-oxo-1H-pyrimidin-5-yl)carbamoylamino]-3-fluoro-pyridine-2-carboxylic acid

Methyl 5-amino-3-fluoro-pyridine-2-carboxylate (500 mg, 2.9 mmol) was added to a stirred solution of (4-nitrophenyl) carbonochloridate (590 mg, 2.9 mmol) in dry MeCN (20 mL) at 0°C. The reaction was stirred for 10 min in a sealed tube. The temperature was increased to 85°C and the reaction was stirred at this temperature for 4 h. A second solution was made by adding 5M NaOH (3.53 mL) to a water (15 mL) suspension of 2,4,5-triamino-1H-pyrimidin-6-one x H_2SO_4 (1.4 g, 5.9 mmol). The hot organic mixture (with solid) was added to the stirred water solution over a period of 2 min at RT. The mixture was stirred at RT for 20 min and the product was collected by filtration. The material was washed with water (15 mL) and MeCN (8 mL). This gave the intermediate ester as a wet solid. LCMS $[M+H]^+$ *m/z* 338. The material was added water (15 mL) and 5M NaOH (3.15 mL) and the reaction was stirred for 15 min at RT. Small residues of solid particles were removed by filtration. The pH of the filtrate was adjusted to ~2 by adding 12M HCl (1 mL) followed by 1M HCl. The product was collected by filtration, washed with water (10 mL) and MeCN (20 mL). The material was dried in a vacuum oven (40°C). Gave 5-[(2,4-diamino-6-oxo-1H-pyrimidin-5-yl)carbamoylamino]-3-fluoro-pyridine-2-carboxylic acid (670 mg, 71%). LCMS $[M+H]^+$ *m/z* 324; ^1H NMR (400 MHz, DMSO-d_6) δ ppm 5.99 (br. s., 2 H) 6.19 (br. s., 2 H) 6.99 (br. s., 1 H) 8.05 (d, *J*=12.32 Hz, 1 H) 8.44 (br. s., 1 H) 9.52 (br. s., 1 H) 9.99 (br. s., 1 H) 12.98 (br. s., 1 H).

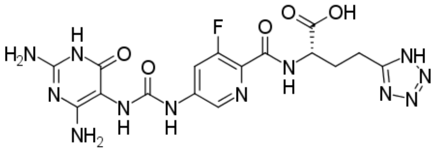

(2S)-2-[[5-[(2,4-diamino-6-oxo-1H-pyrimidin-5-yl)carbamoylamino]-3-fluoro-pyridine-2-carbonyl]amino]-4-(1H-tetrazol-5-yl)butanoic acid (TH9975)

5-[(2,4-diamino-6-oxo-1H-pyrimidin-5-yl)carbamoylamino]-3-fluoro-pyridine-2-carboxylic acid (450 mg, 1.4 mmol), (2S)-2-amino-4-[1-(2-cyanoethyl)-1H-1,2,3,4-tetrazol-5-yl]butanoate hydrochloride (530 mg, 1.9 mmol) (synthesis has previously been described in *Chem. Pharm. Bull. 43(2) 230-235 (1995)*), EDCI (400 mg, 2.1 mmol), HOBt (282 mg, 2.1 mmol), N,N-diisopropylethylamine (1.2 mL, 7.0 mmol) and DMSO (15 mL) was stirred at RT over night. Residues of solid particles were removed by filtration and the filtrate was purified by acidic prep-HPLC. Gave the intermediate ester/nitrile (170 mg). LCMS $[M+H]^+$ *m/z* 544. The material was suspended in water (1 mL) and 5M NaOH (0.24 mL) was added. The reaction was stirred at RT for 20 min and at 50°C for 40 min. The pH was adjusted to ~3 with 2M HCl.

The product was collected by filtration, washed with water (4 mL) and dried in a vacuum oven (40°C) over night. Gave (2S)-2-[[5-[(2,4-diamino-6-oxo-1H-pyrimidin-5-yl)carbamoylamino]-3-fluoro-pyridine-2-carbonyl]amino]-4-(1H-tetrazol-5-yl)butanoic acid (115 mg, 17%). LCMS $[M+H]^+$ *m/z* 477; ^{1}H NMR (400 MHz, DMSO-d_6) δ ppm 2.14 - 2.42 (m, 2 H) 2.85 - 3.03 (m, 2 H) 4.40 - 4.54 (m, 1 H) 5.99 (br. s., 2 H) 6.19 (br. s., 2 H) 6.97 (br. s., 1 H) 8.04 (d, *J*=13.3 Hz, 1 H) 8.47 (br. s., 1 H) 8.68 (d, *J*=8.1 Hz, 1 H) 9.50 (br. s., 1 H) 9.99 (br. s., 1 H) 12.89 (br. s., 1 H) 15.98 (br. s., 1 H).

Supplementary Discussion

In the context of HPLM, it has been suggested that hypoxanthine can exert toxicity by inhibiting uridine monophosphate synthase (UMPS).[32] We thus speculated that UMPS inhibition might also contribute to the observed MTHFD inhibitor toxicity that is exacerbated in presence of hypoxanthine (Extended Data Fig. 5e). To test this hypothesis, we investigated whether uric acid or a UMPS inhibitor (pyrazofurin) could mimic hypoxanthine sensitization, and whether hypoxanthine sensitization could be reversed with xanthine oxidase inhibitors (allopurinol and febuxostat) or deoxyuridine. However, 350 µM uric acid had no effect on TH9619 toxicity (Extended Data Fig. 5f) and while pyrazofurin displayed a small additive effect with TH9619, the sensitization did not compare to the effect of hypoxanthine (Extended Data Fig. 5g). Moreover, deoxyuridine did not rescue toxicity, the UMPS substrate orotate had no effect (Fig. 5h) and allopurinol and febuxostat did not reverse hypoxanthine sensitization (Fig. 5i). We also proposed that production of H_2O_2 may contribute to toxicity via induction of reactive oxygen species through hypoxanthine conversion to uric acid, however, 100 µM H_2O_2 had no effect on TH9619 toxicity (Extended Data Fig. 5j). Finally, no increase in uric acid concentrations following addition of hypoxanthine was observed (Extended Data Fig. 5k).

Supplementary Figure 1

a

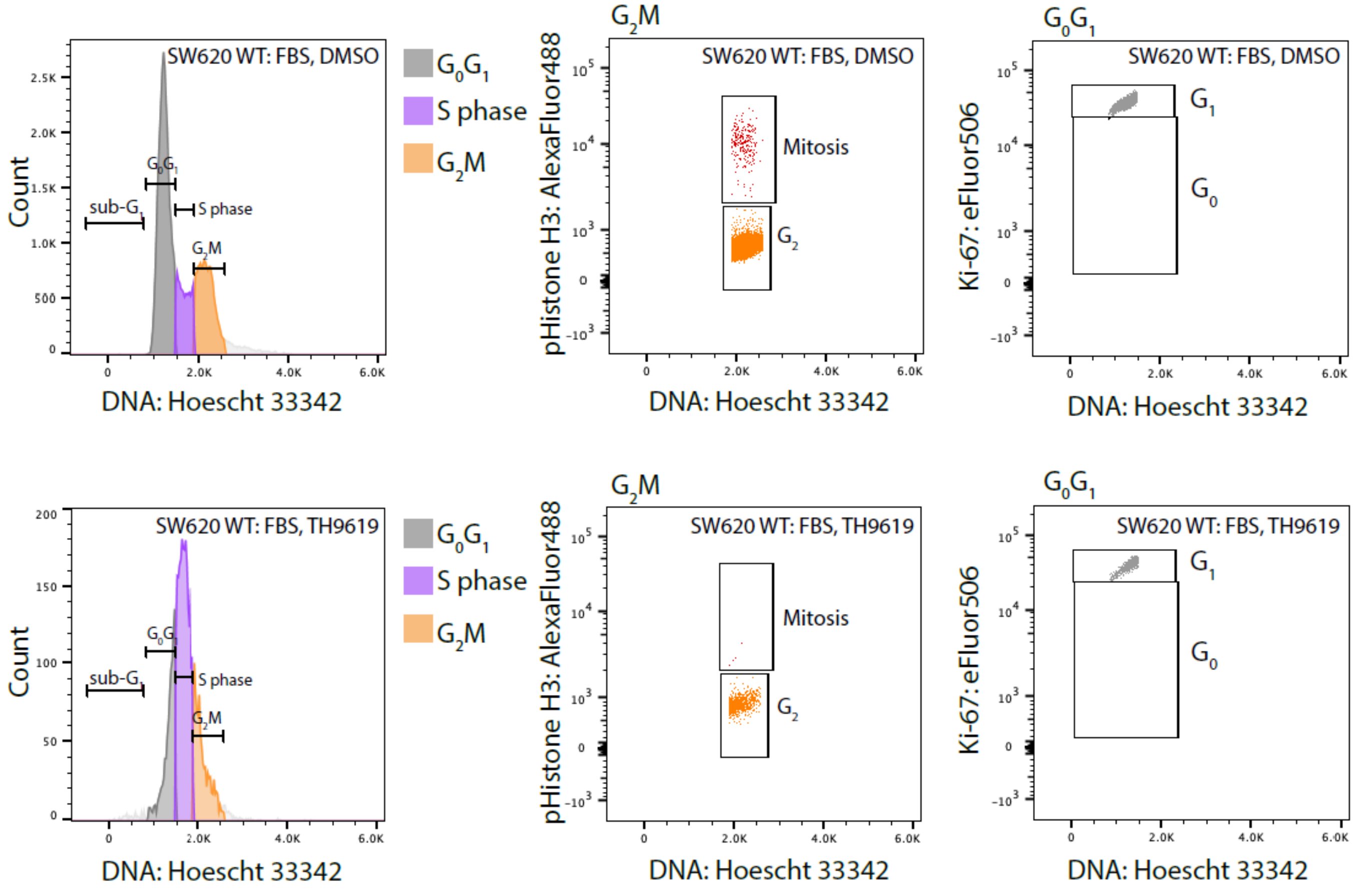

b

SW620 WT untreated Control
Annexin-V PI

Gate	%Parent	Cell Count
All	100	50000
Total	91.5	45742
Single Cells	91.1	41666

Gate	Staining	%Parent
Q1	PI	0.0096
Q2	AnnexinV/PI	0.58
Q3	AnnexinV	0.91
Q4	Viable	98.5

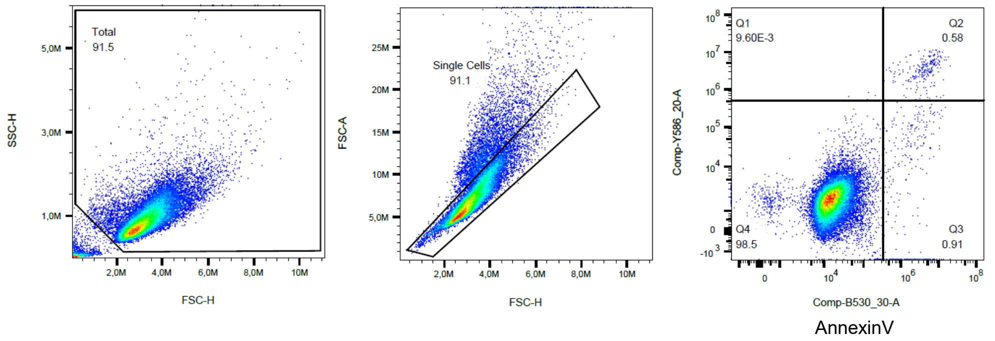

SW620 WT 24h TH9619
Annexin-V PI

Gate	%Parent	Cell Count
All	100	50000
Total	64.6	32288
Single Cells	94.1	30392

Gate	Staining	%Parent
Q1	PI	0.14
Q2	AnnexinV/PI	9.36
Q3	AnnexinV	11.3
Q4	Viable	79.2

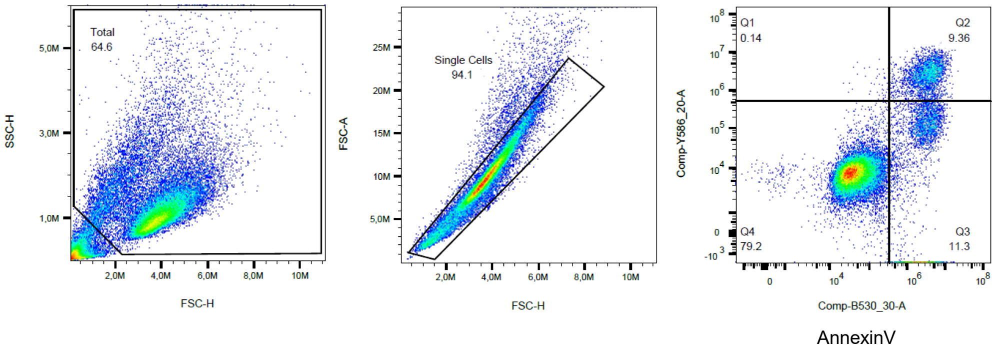

Supplementary Figure 1. Flow Cytometry Gating Strategy.
General gating strategy for all analyses: 1) Exclusion of debris by forward (FSC) and side (SSC) scatter. 2) Removal of cell doublets and other cell aggregates by either time-of-flight (TOF) gating (for cell cycle analysis) or FSC-A vs FSC-H gating (for apoptosis analysis).
(a) Cell cycle analysis was determined by DNA content analysis using Hoescht33342 (distinguishing cells in G_0G_1, S phase and G_2M). Cells were then separated in to G_1 (Ki-67+) and G_0 (Ki-67-) and mitotic cells were identified by expression of phosphor-histone H3.
(b) Apoptotic cell death was determined using AnnexinV and PI double staining. Cells were then separated using a quadrant gate into four subpopulations PI+, AnnexinV+/PI+, AnnexinV+ and viable (AnnexinV-/PI-) cell populations for analysis. For (a) and (b) quantitative and statistical analysis was performed on these distinct cell populations. An exemplary gating scheme is given above for each type of flow cytometry experiment performed in this study.
