# Supplementary material for: Formate overflow drives toxic folate trapping in MTHFD1 inhibited cancer cells
Source: Nat Metab. 2023 Apr 3;5(4):642–59. doi: 10.1038/s42255-023-00771-5 (PMC10132981; doi:10.1038/s42255-023-00771-5)
Supplement: Supplementary file 2 — Reporting Summary [file 42255_2023_771_MOESM2_ESM.pdf]

nature portfolio

Corresponding author(s): Johannes Meiser

Last updated by author(s): Feb 13, 2023

Reporting Summary

Nature Portfolio wishes to improve the reproducibility of the work that we publish. This form provides structure for consistency and transparency in reporting. For further information on Nature Portfolio policies, see our Editorial Policies and the Editorial Policy Checklist.

Statistics

For all statistical analyses, confirm that the following items are present in the figure legend, table legend, main text, or Methods section.

n/a	Confirmed	
☐	☒	The exact sample size (*n*) for each experimental group/condition, given as a discrete number and unit of measurement
☐	☒	A statement on whether measurements were taken from distinct samples or whether the same sample was measured repeatedly
☐	☒	The statistical test(s) used AND whether they are one- or two-sided<br>*Only common tests should be described solely by name; describe more complex techniques in the Methods section.*
☒	☐	A description of all covariates tested
☐	☒	A description of any assumptions or corrections, such as tests of normality and adjustment for multiple comparisons
☐	☒	A full description of the statistical parameters including central tendency (e.g. means) or other basic estimates (e.g. regression coefficient) AND variation (e.g. standard deviation) or associated estimates of uncertainty (e.g. confidence intervals)
☐	☒	For null hypothesis testing, the test statistic (e.g. *F*, *t*, *r*) with confidence intervals, effect sizes, degrees of freedom and *P* value noted<br>*Give P values as exact values whenever suitable.*
☒	☐	For Bayesian analysis, information on the choice of priors and Markov chain Monte Carlo settings
☒	☐	For hierarchical and complex designs, identification of the appropriate level for tests and full reporting of outcomes
☒	☐	Estimates of effect sizes (e.g. Cohen's *d*, Pearson's *r*), indicating how they were calculated

Our web collection on statistics for biologists contains articles on many of the points above.

Software and code

Policy information about availability of computer code

Data collection	Xcalibur 4.4.16.14 (Thermo Fisher), ImageStudio 5.2 (LI-COR Biosciences), NovoExpress 1.5.0 (Agilent), IncuCyte ZOOM 2021C and 2018A (Essen Bioscience), BD FACSDiva 8.0.1 (BD Bioscience), Hidex Sense Software 1.0 (Hidex Oy), Cytation 5 (Agilent)
Data analysis	TraceFinder 4.1 (Thermo Fisher), ImageStudio 5.2 (LI-COR Biosciences), FlowJo 10.6.2 or 10.7.2 (BD Biosciences), GraphPad Prism Vers9, IncuCyte ZOOM 2021C and 2018A (Essen Bioscience), ImageJ Fiji 2.5.0 (National Institutes of Health, MD, USA), Skyline software (21.2), CellProfiler, CRISPResso2 online tool

For manuscripts utilizing custom algorithms or software that are central to the research but not yet described in published literature, software must be made available to editors and reviewers. We strongly encourage code deposition in a community repository (e.g. GitHub). See the Nature Portfolio guidelines for submitting code & software for further information.

Data

Policy information about availability of data

All manuscripts must include a data availability statement. This statement should provide the following information, where applicable:

- Accession codes, unique identifiers, or web links for publicly available datasets
- A description of any restrictions on data availability
- For clinical datasets or third party data, please ensure that the statement adheres to our policy

All data generated or analyzed during the present study, including source data, can be found in the article, Extended Data or Supplementary Information. Source data are provided with this paper.

Human research participants

Policy information about studies involving human research participants and Sex and Gender in Research.

Reporting on sex and gender	N/A
Population characteristics	N/A
Recruitment	N/A
Ethics oversight	N/A

Note that full information on the approval of the study protocol must also be provided in the manuscript.

Field-specific reporting

Please select the one below that is the best fit for your research. If you are not sure, read the appropriate sections before making your selection.

☒ Life sciences ☐ Behavioural & social sciences ☐ Ecological, evolutionary & environmental sciences

For a reference copy of the document with all sections, see nature.com/documents/nr-reporting-summary-flat.pdf

Life sciences study design

All studies must disclose on these points even when the disclosure is negative.

Sample size	For flow cytometry, between 10,000 and 30,000 single cell events were acquired and quantified per replicate and condition following the guidelines by Cossarizza et al., 2019 (Guidelines for the use of flow cytometry and cell sorting in immunological studies - 2nd edition). Unless otherwise stated, n=3 was chosen as the minimal number of biological replicates per experiment that would allow for adequate analysis to draw meaningful conclusions of the data. We determined this to be sufficient based on the low observed variability between samples from these cell culture in vitro experiments.
Data exclusions	No data were excluded from analysis.
Replication	All experiments in this study were performed at least twice under independent experimental conditions. All attempts at replication have been successful. To ensure reproducibility, same reagent suppliers and catalog numbers were maintained as long as possible at all concerned experimental locations. When new reagents needed to be used, validation and optimization experiments were conducted to ensure comparable results.
Randomization	In vitro experiments were not randomized, but independent experiments were for example performed in different formats and by different investigators as mitigiation measures to cancel out experimental bias. Also, cells were randomly seeded from one cell population and plate lanes were randomly allocated to condtions. All Mass Spec samples were randomized during analysis to avoid bias from e.g. instrument drift.
Blinding	Blinding was not possible for in vitro experiments as treatment conditions were evident from the data. Quantifications were performed using computational pipelines (FlowJo for flow cytometry analysis, Tracefinder and MetaboliteDetector for Mass Spectrometry analysis).

Reporting for specific materials, systems and methods

We require information from authors about some types of materials, experimental systems and methods used in many studies. Here, indicate whether each material, system or method listed is relevant to your study. If you are not sure if a list item applies to your research, read the appropriate section before selecting a response.

Materials & experimental systems

n/a	Involved in the study	
☐	☒	Antibodies
☐	☒	Eukaryotic cell lines
☒	☐	Palaeontology and archaeology
☒	☐	Animals and other organisms
☒	☐	Clinical data
☒	☐	Dual use research of concern

Methods

n/a	Involved in the study	
☒	☐	ChIP-seq
☐	☒	Flow cytometry
☒	☐	MRI-based neuroimaging

Antibodies

Antibodies used

Anti-β-Actin (8H10D10) (Cell Signaling Technology Cat#3700; RRID:AB_2242334); Anti-MTHFD2 (Abcam Cat#ab56772; RRID:AB_2147537); Anti-MTHFD2 (D8W9U) (Cell Signaling Technology Cat#41377; RRID:AB_2799200); anti-MTHFD1 (Atlas Antibodies Cat#HPA000704; RRID:AB_1079424); Anti-MTHFD1 (Thermo Fisher Scientific Cat#PA5-42825; RRID:AB_2609491); MTHFD1L (Proteintech Cat.#16113-1-AP; RRID:AB_2250974); Anti-SHMT1 (Sigma-Aldrich Cat#HPA023314; RRID:AB_1856830); Anti-SHMT2 (Sigma-Aldrich Cat#HPA020549; RRID:AB_1856834); anti-HSP60 (Abcam Cat#ab46798; RRID:AB_881444); anti-Vinculin (Cell Signaling Technology Cat#4650; RRID:AB_10559207); anti-Lamin A/C (Cell Signaling Technology Cat#2032; RRID:AB_2136278); anti-α-Tubulin (Abcam Cat#ab7291; RRID:AB_2241126); anti-ATP synthase beta (Thermo Fisher Scientific Cat#A21351; RRID:AB_221512); anti-TOM20 (Proteintech Cat#11802-1-AP; RRID:AB_2207530); anti-SOD-1 (G-11) (Santa Cruz Biotechnology Cat#sc-17767; RRID:AB_628301); Alexa Fluor® 488 anti-Histone H3 Phospho (Ser10) Antibody (BioLegend Cat#650803; RRID:AB_10917386); eFluor 506 anti-human Ki-67 Antibody (Thermo Fisher Scientific Cat# 69-5698-82; RRID:AB_11151689); Goat Anti-Mouse IgG (H+L) (IRDye® 680RD) (LI-COR Biosciences Cat#926-68070; RRID:AB_10956588); Donkey Anti-Rabbit IgG (H+L) (IRDye® 800CW) (LI-COR Biosciences Cat#926-32213; RRID:AB_621848); Donkey Anti-Mouse IgG (H+L) (IRDye® 800CW) (LI-COR Biosciences Cat#926-32212; RRID:AB_621847); Peroxidase-AffiniPure Donkey Anti-Rabbit IgG (H+L) (Jackson ImmunoResearch Cat#711-035-152; RRID:AB_10015282); Peroxidase-AffiniPure Donkey Anti-Mouse IgG (H+L) (Jackson ImmunoResearch Cat#715-035-150; RRID:AB_234077).

Validation

All antibodies have been validated for use as stated on the manufacturer's product page:
Anti-β-Actin (8H10D10): KO validated. Suitable for ICC/IF, WB. Reacts with M, R, B, Dg, H, Mk, Hm. HeLa, Jurkat, NIH/3T3, PC-12, Rat2, CHO, MDBK and MDCK (DOx-inducible b-actin) whole cell lysates were used as positive controls.
Anti-MTHFD2 (Abcam): KO validated. Suitable for WB, IHC-P, Flow Cyt. Reacts with H. HEK293T, HepG2 and HeLa whole cell lysates were used as positive controls. In-house validation for IF and WB using siRNA KD and CRISPR KO.
Anti-MTHFD2 (D8W9U): Suitable for WB, IP. Reacts with H. HeLa and 293 whole cell lysates were used as positive control. In-house validation for WB using CRISPR KO.
anti-MTHFD1 (Atlas): Suitable for WB, IHC. Reacts with H, M, R. Validated with siRNA KD in A-431 whole cell lysates. In-house validation for WB using CRISPR KO.
anti-MTHFD1 (ThermoFisher): Suitable for WB, IHC. Reacts with H. Hep-G2 whole cell lysates were used as positive controls. In-house validation for WB using CRISPR KO.
Anti-MTHFD1L: Suitable for WB, IP, IHC, IF. Reacts with H, M. HEK-293, COLO 320, mouse ovary tissue, HeLa, HepG2 lysates were used as positive controls. In-house validation for WB using CRISPR KO and shRNA KD.
Anti-SHMT1: Suitable for WB, IHC. Reacts with H. U2-OS cells, human liver, testis and kidney tissue were used as positive control. In-house validation for WB using CRISPR KO.
Anti-SHMT2: Suitable for WB, IHC. Reacts with H, M, R. RH-30, NIH3T3, NBT-II, A-431 and U251 MG cells were used as positve control. In-house validation for WB using CRISPR KO.
anti-HSP60: Suitable for WB, IHC, IF, Flow Cyt, IP. Reacts with M, R, H, P. T47-D, MCF6, SW480 whole cell lysates were used as positive control.
anti-Vinculin: Suitable for WB. Reacts with H, M, R, Mk, Dg. HeLa, IGROV1, HepG2, RD, COS and MDCK whole cell lysates were used as positive control.
anti-Lamin A/C: Suitable for WB, IHC. Reacts with H, M, R. HeLa, Raw 264.7 and PC12 whole cell lysates were used as positive control. In-frame truncation mutation of lamin-encoding gene LMNA in HeLa cells was used for validation.
anti-α-Tubulin: Suitable for Flow Cyt, IF, IHC, WB. Reacts with M, R, H. HeLa, PC12, SV40LT-SMC, NIH/3T3, Rat liver tissue, Rat heart tissue lysate was used as positive control.
anti-ATP synthase beta: Suitable for WB, IF. Reacts with B, H, M, Mk, R. HepG2, SK-Ov-3, MDA-MB-231 were used as positive controls. Validated with siRNA KD in HepG2 cells.
anti-TOM20: Suitable for WB, IP, IHC, IF, Flow Cyt, ELISA. Reacts with H, M, R, B, C, Hm, Mk, P. HEK-293, HeLa, SH-SY5Y, mouse kidney tissue, rat kidney tissue lysates were used as positive control.
anti-SOD-1 (G-11): Suitable for WB, IP, IF, IHC, ELISA. Reacts with H. Jurkat, DU 145 an HEK293T whole cell lysates were used as positive controls.
Alexa Fluor® 488 anti-Histone H3 Phospho (Ser10): Suitable for Flow Cyt. Reacts with H. Validated in Nocodazole-treated HeLa cells.
eFluor 506 anti-human Ki-67: Suitable for Flow Cyt. Reacts with H, M, R. Validated with anti-mouse CD3e stimulated vs unstimulated mouse splenocytes.

Eukaryotic cell lines

Policy information about cell lines and Sex and Gender in Research

Cell line source(s)

SW620, HCT116, MDA-MB-468, MDA-MB-231 cells (ATCC), PA-TU-8988T cells (DSMZ). SW620 MTHFD2-/- and SHMT1-/- pools were purchased from Synthego. SW620 MTHFD2-/- and SHMT1-/- clones were generated from pools provided by Synthego, after validation of target deletion, early passage clonal cell lines were used in experiments. MDA-MB-468 MTHFD2-/- cells were generated and validated as described previously (Kiweler et al. 2022; 10.1038/s41467-022-30363-y).

	Optimization experiments were performed in 3-4 clones, and then one clone was selected for use in experiments across each location.
Authentication	SW620 and HCT116 cells were authenticated by STR analysis, frozen down and these stocks were used in experiments. PA-TU-8988T cells used in this study were newly purchased from DSMZ and the authenticity was confirmed by STR analysis by the supplier prior purchasing. These cells were cultured and expanded from the original vial obtained from the supplier frozen down and used in experiments.
Mycoplasma contamination	Cell lines were routinely tested for mycoplasma contamination using the MycoAlertTM Mycoplasma Detection Kit (Lonza). All cell lines in this study tested negative for mycoplasma contamination.
Commonly misidentified lines (See ICLAC register)	None of the cell lines used in this study are listed in the ILAC register of commonly misidentified cell lines.

Flow Cytometry

Plots

Confirm that:

☒ The axis labels state the marker and fluorochrome used (e.g. CD4-FITC).

☒ The axis scales are clearly visible. Include numbers along axes only for bottom left plot of group (a 'group' is an analysis of identical markers).

☒ All plots are contour plots with outliers or pseudocolor plots.

☒ A numerical value for number of cells or percentage (with statistics) is provided.

Methodology

Sample preparation	For Cell Cycle Analysis: Adherent cells were collected, washed and resuspended in PBS. Cells were resuspended in fixation/ permeabilization buffer and washed in permeabilization buffer (Thermo Fisher Scientific). Cells were stained with Ki-67 eFluor 506 (1:20, BioLegend) and p-Histone H3 AlexaFluor488 (1:50, BioLegend) or isotype controls in permeabilization buffer at 4°C in the dark. Cells were washed twice in permeabilization buffer then resuspended in 100 µl PBS containing 10 ug/ml Hoechst 33342 (Life Technologies) and 100 ug/ml RNaseA5 (Thermo Scientific) and incubated for 15 minutes at room temperature in the dark. Samples were analyzed on a BD LSRII (BD Biosciences) and data analyzed in FlowJo (10.7.2, BD).<br><br>For Cell Death Analysis: Adherent cells were washed with PBS. PBS fraction was collected and cells were detached with trypsin. Detached cells were collected in medium. All collected fractions were combined and centrifuged. The pellet was washed with PBS and resuspended in 50 µl AnnexinV-FITC staining solution (5% AnnexinV-FITC in AnnexinV binding buffer (10 mM HEPES pH 7.4, 140 mM NaCl, 2.5 mM CaCl2, 0.1% BSA in ddH2O)) and incubated for 15 min on ice in the dark. 450 µl PI-staining solution (1.1 µg/ml PI in AnnexinV binding buffer) was added immediately prior to measurement using NovoCyte Quanteon (Agilent) and NovoExpress software (Agilent, version 1.5.0). Analysis was performed in FlowJo Version 10.6.2 (BD).
Instrument	NovoCyte Quanteon (4025) (Agilent); BD LSR II 4 laser (BD Bioscience)
Software	Data aquisition: BD FACSDiva 8.0.1 (BD Bioscience) or NovoExpress 1.5.0 (Agilent); Data Analysis: FlowJo Software (Vers 10.6.2 or 10.7.2) (BD Biosciences)
Cell population abundance	A minimum of 10,000 events were acquired per sample following debris and doublet exclusion. Positive populations were determined using single-stained negative (unstained or isotype) and positive controls (e.g. heat-treated cell population for cell death analysis).
Gating strategy	FSC/SSC gating to exclude debris using unstained control (debris defined as small FSC vs. SSC). Doublets and cell aggregates were excluded by gating of FSC-A/FSC-H (for cell death analysis) or FSC/TOF (for cell cycle analysis).

☒ Tick this box to confirm that a figure exemplifying the gating strategy is provided in the Supplementary Information.
