# Supplementary material for: Formate overflow drives toxic folate trapping in MTHFD1 inhibited cancer cells
Source: Nat Metab. 2023 Apr 3;5(4):642–59. doi: 10.1038/s42255-023-00771-5 (PMC10132981; doi:10.1038/s42255-023-00771-5)

Source Data - Unprocessed images of Western blots related to Figure 1j.

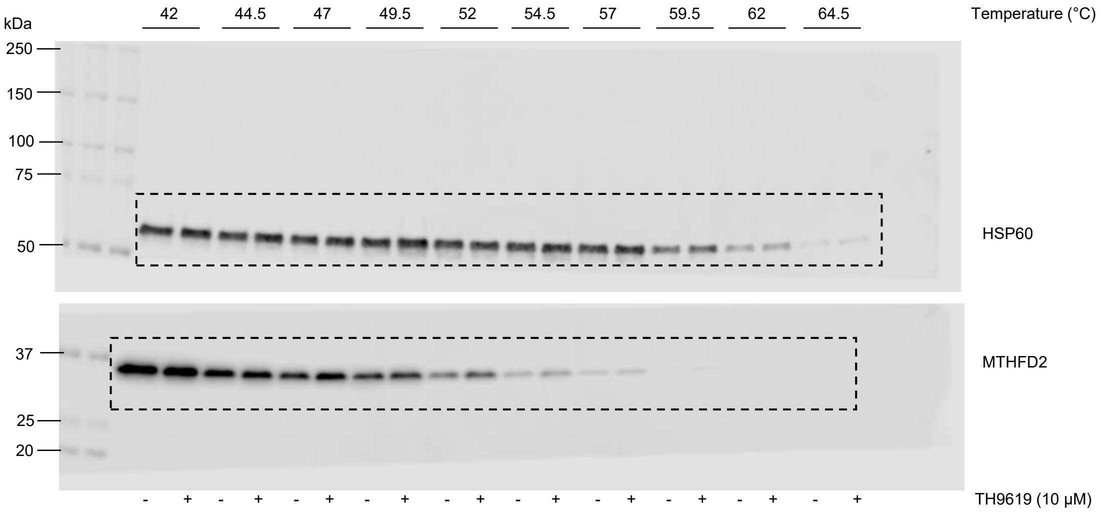

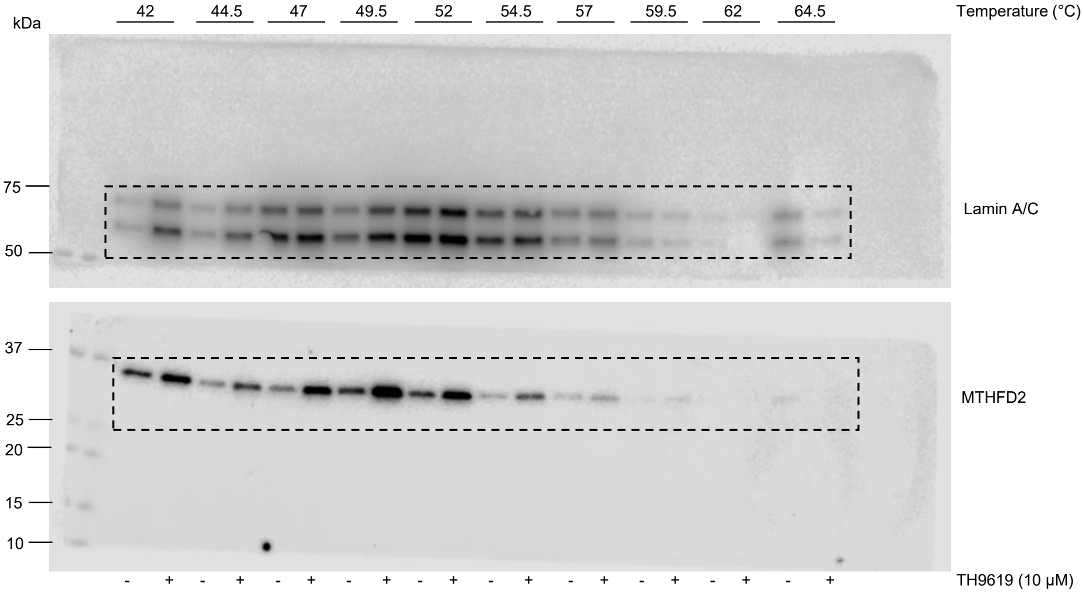

Source Data - Unprocessed images of Western blots related to Figure 1k.

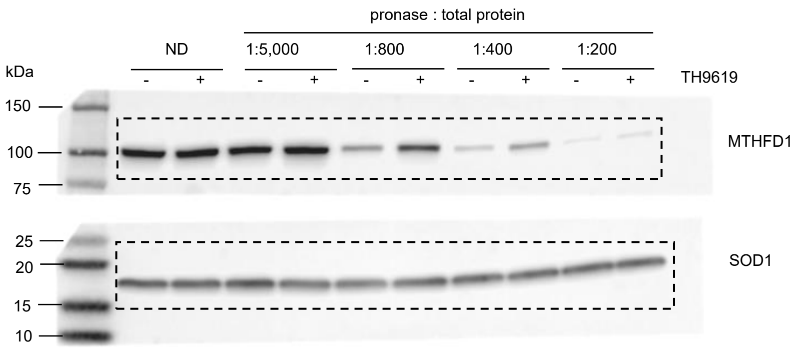

Supplement: Source Data Fig. 1 — Unprocessed western blots. [file 42255_2023_771_MOESM6_ESM.pdf]
