# Supplementary figures and images for: Formate overflow drives toxic folate trapping in MTHFD1 inhibited cancer cells
Source: Nat Metab. 2023 Apr 3;5(4):642–59. doi: 10.1038/s42255-023-00771-5 (PMC10132981; doi:10.1038/s42255-023-00771-5)

Source Data - Unprocessed images of Western blots related to Extended Data Figure 6 b,c.

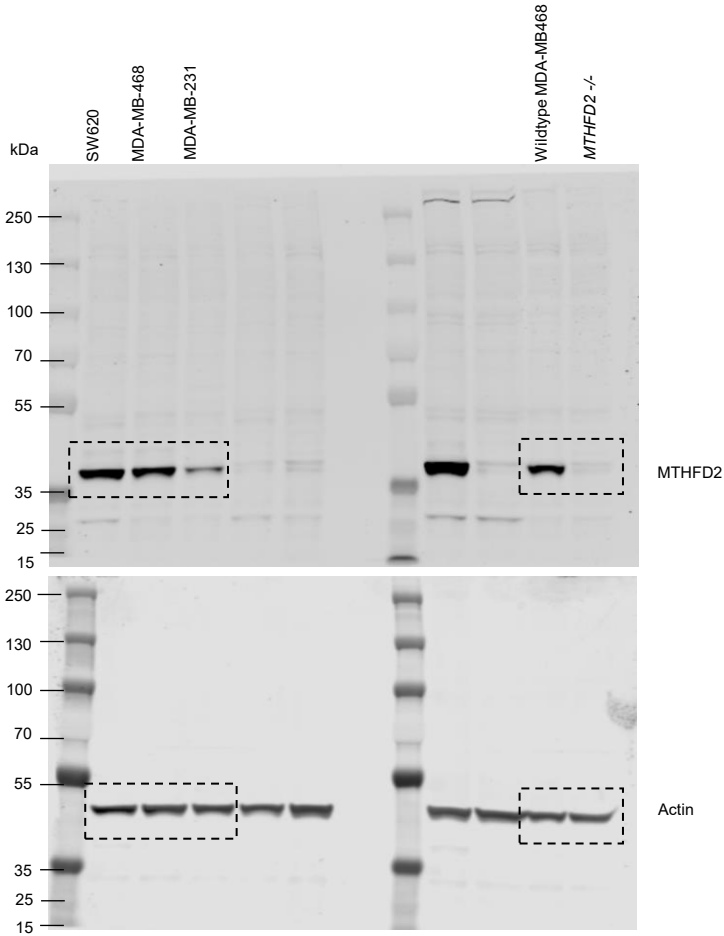

Supplement: Source Data Extended Data Fig. 6 — Unprocessed western blots. [file 42255_2023_771_MOESM20_ESM.pdf]
